# Characterization of the ADP-*β*-d-*manno*-heptose biosynthetic enzymes from two pathogenic *Vibrio* strains

**DOI:** 10.1007/s00253-024-13108-3

**Published:** 2024-03-18

**Authors:** Zhaoxiang Shi, Yue Tang, Zhenyi Wang, Min Wang, Zijian Zhong, Jingming Jia, Yihua Chen

**Affiliations:** 1 School of Traditional Chinese Materia Medica, Shenyang Pharmaceutical University, Shenyang, 117004 China; 2 State Key Laboratory of Microbial Resources, Institute of Microbiology, Chinese Academy of Sciences, Beijing, 100101 China; 3 RDFZ Xishan School, Beijing, 100193 China; 4 School of Biotechnology and Health Sciences, Wuyi University, Jiangmen, 529020 China; 5 University of Chinese Academy of Sciences, Beijing, 100049 China

**Keywords:** ADP-*β*-d-*manno*-heptose biosynthesis, *Vibrio* strains, Nucleotidyltransferase, Kinetic analysis, Lipopolysaccharide

## Abstract

**Abstract:**

ADP-activated *β*-d-*manno*-heptoses (ADP-*β*-d-*manno*-heptoses) are precursors for the biosynthesis of the inner core of lipopolysaccharide in Gram-negative bacteria. Recently, ADP-d-*glycero*-*β*-d-*manno*-heptose (ADP-d,d-*manno*-heptose) and its C-6′′ epimer, ADP-l-*glycero*-*β*-d-*manno*-heptose (ADP-l,d-*manno*-heptose), were identified as potent pathogen-associated molecular patterns (PAMPs) that can trigger robust innate immune responses. Although the production of ADP-d,d-*manno*-heptose has been studied in several different pathogenic Gram-negative bacteria, current knowledge of ADP-*β*-d-*manno*-heptose biosynthesis in *Vibrio* strains remains limited. Here, we characterized the biosynthetic enzymes of ADP-d,d-*manno*-heptose and the epimerase that converts it to ADP-l,d-*manno*-heptose from *Vibrio cholerae* (the causative agent of pandemic cholera) and *Vibrio parahaemolyticus* (non-cholera pathogen causing vibriosis with clinical manifestations of gastroenteritis and wound infections) in comparison with their isozymes from *Escherichia coli*. Moreover, we discovered that *β*-d-mannose 1-phosphate, but not *α*-d-mannose 1-phosphate, could be activated to its ADP form by the nucleotidyltransferase domains of bifunctional kinase/nucleotidyltransferases HldE_VC_ (from *V. cholerae*) and HldE_VP_ (from *V. parahaemolyticus*). Kinetic analyses of the nucleotidyltransferase domains of HldE_VC_ and HldE_VP_ together with the *E. coli*–derived HldE_EC_ were thus carried out using *β*-d-mannose 1-phosphate as a mimic sugar substrate. Overall, our works suggest that *V. cholerae* and *V. parahaemolyticus* are capable of synthesizing ADP-*β*-d-*manno*-heptoses and lay a foundation for further physiological function explorations on *manno*-heptose metabolism in *Vibrio* strains.

**Key points:**

*• Vibrio strains adopt the same biosynthetic pathway as E. coli in synthesizing ADP-β-*d*-manno-heptoses.*

*• HldEs from two Vibrio strains and E. coli could activate β-* d *-mannose 1-phosphate to ADP-β-* d *-mannose.*

*• Comparable nucleotidyltransfer efficiencies were observed in the kinetic studies of HldEs.*

**Supplementary Information:**

The online version contains supplementary material available at 10.1007/s00253-024-13108-3.

## Introduction

ADP-activated *β*-d-*manno*-heptoses (ADP-*β*-d-*manno*-heptoses), including ADP-d-*glycero*-*β*-d-*manno*-heptose (ADP-d,d-*manno*-heptose) and ADP-l-*glycero*-*β*-d-*manno*-heptose (ADP-l,d-*manno*-heptose), are common building blocks of lipopolysaccharides (LPSs) in Gram-negative bacteria. In some cases, blockade of *β*-d-*manno*-heptose biosynthesis can remarkably increase bacterial sensitivity to antibiotics and reduce bacterial virulence (Raetz and Whitfield 2002). In addition, ADP-activated *β*-d-*manno*-heptoses are sugar donors of heptosylation of bacterial autotransporters that deliver virulence factors to the bacterial surface (Lu et al. 2014). They are also key intermediates of the biosynthesis of nucleoside antibiotics like septacidins and spicamycins with fascinating antifungal and antitumor bioactivities (Guo et al. 2021; Tang et al. 2018). Recently, ADP-*β*-d-*manno*-heptoses from several pathogenic bacteria like *Yersinia pseudotuberculosis* and *Campylobacter jejuni* were identified as potent pathogen-associated molecular patterns (PAMPs), which can be sensed by alpha-protein kinase 1 (ALPK1) and trigger robust innate immune responses via nuclear factor kappa-B (NF-*κ*B) signaling pathway (Zhou et al. 2018; Cui et al. 2021). Moreover, two intermediates of ADP-*β*-d-*manno*-heptose biosynthesis, d-*glycero*-*β*-d-*manno*-heptose 1,7-bisphosphate (HBP) and d-*glycero*-*β*-d-*manno*-heptose 1-phosphate (H1P), were proposed to be PAMPs that can elicit NF-*κ*B-mediated innate immune responses during studies on pathogens like *Helicobacter pylori* and *Neisseria meningitides* (Gaudet et al. 2015; Malott et al. 2013; Zimmermann et al. 2017; Garcia-Weber and Arrieumerlou 2021), underlining the immunological roles of the *β*-d-*manno*-heptose metabolites.

**Fig. 1:**
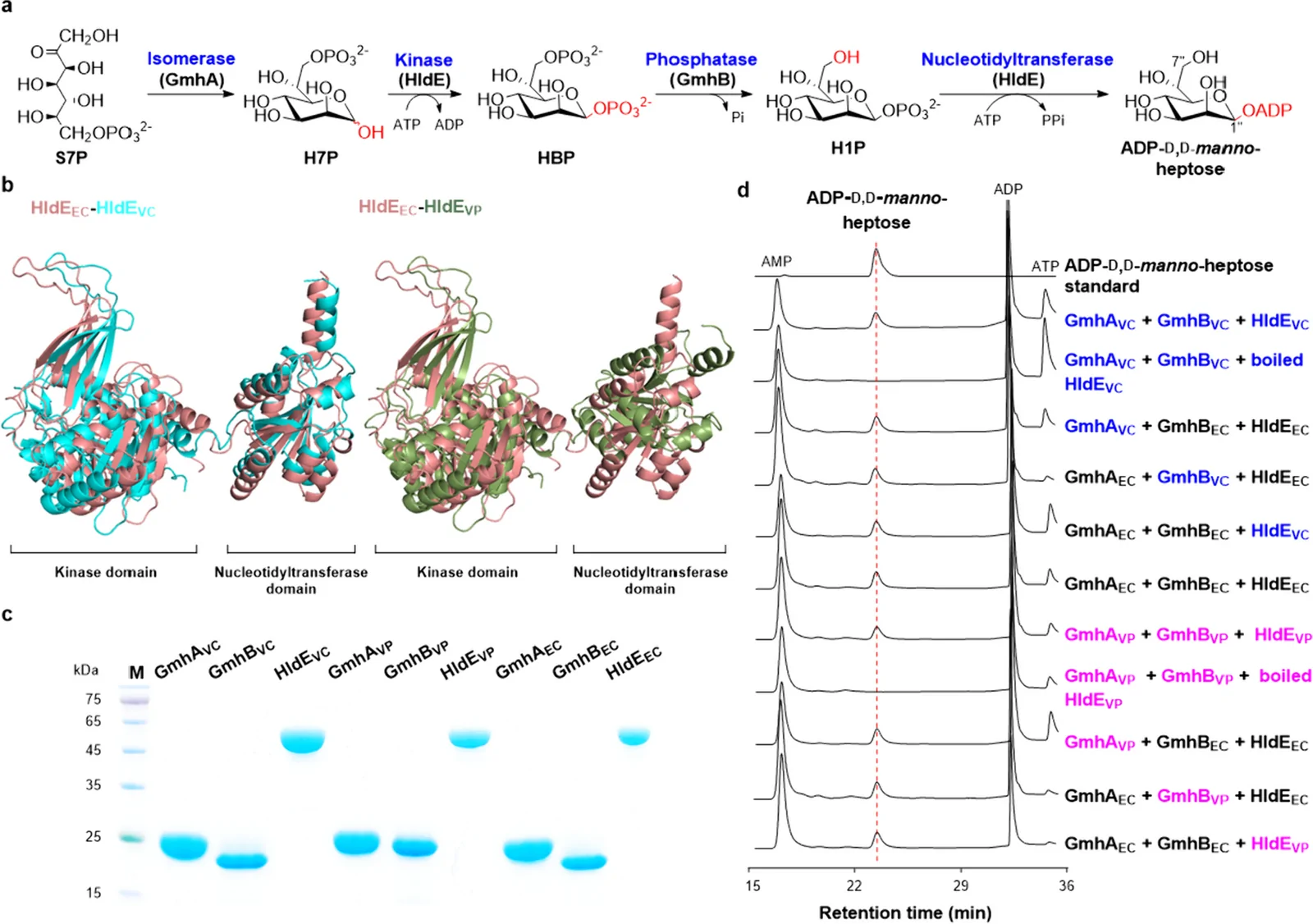
Characterization of the biosynthetic enzymes of ADP-d,d*-manno*-heptose from two pathogenic *Vibrio* strains. **a** The biosynthetic pathway of ADP-d,d*-manno*-heptose. S7P, d-sedoheptulose 7-phosphate; H7P, d-*glycero*-*β-*d-*manno*-heptose 7-phosphate; HBP, d-*glycero*-*β-*d-*manno*-heptose 1,7-biphosphate; H1P, d-*glycero*-*β-*d-*manno*-heptose 1-phosphate. **b** Structural comparison of bifunctional HldEs from *Vibrio* strains with *E. coli*–derived HldE_EC_. HldE_VC_ and HldE_VP_ are bifunctional kinase/nucleotidyltransferses from *V. cholerae* O1 2010EL-1786 and *V. parahaemolyticus* CGMCC 1.1997, respectively. **c** SDS-PAGE analysis of *N*-His_6_ tagged ADP-d,d*-manno*-heptose biosynthetic enzymes. Lane M, protein marker. GmhA_VC_, GmhB_VC_, and HldE_VC_ are from *V. cholerae* O1 2010EL-1786; GmhA_VP_, GmhB_VP_, and HldE_VP_ are from *V. parahaemolyticus* CGMCC 1.1997; GmhA_EC_, GmhB_EC_, and HldE_EC_ are from *E. coli* BL21. **d** HPLC profiles of the enzymatic assays of the ADP-d,d*-manno*-heptose synthetic enzymes using S7P and ATP as substrates. The detection wavelength was set at 254 nm. Enzymes from *V. cholerae* O1 2010EL-1786, *V. parahaemolyticus* CGMCC 1.1997, and *E. coli* are indicated in blue, rose, and black, respectively

**Fig. 2:**
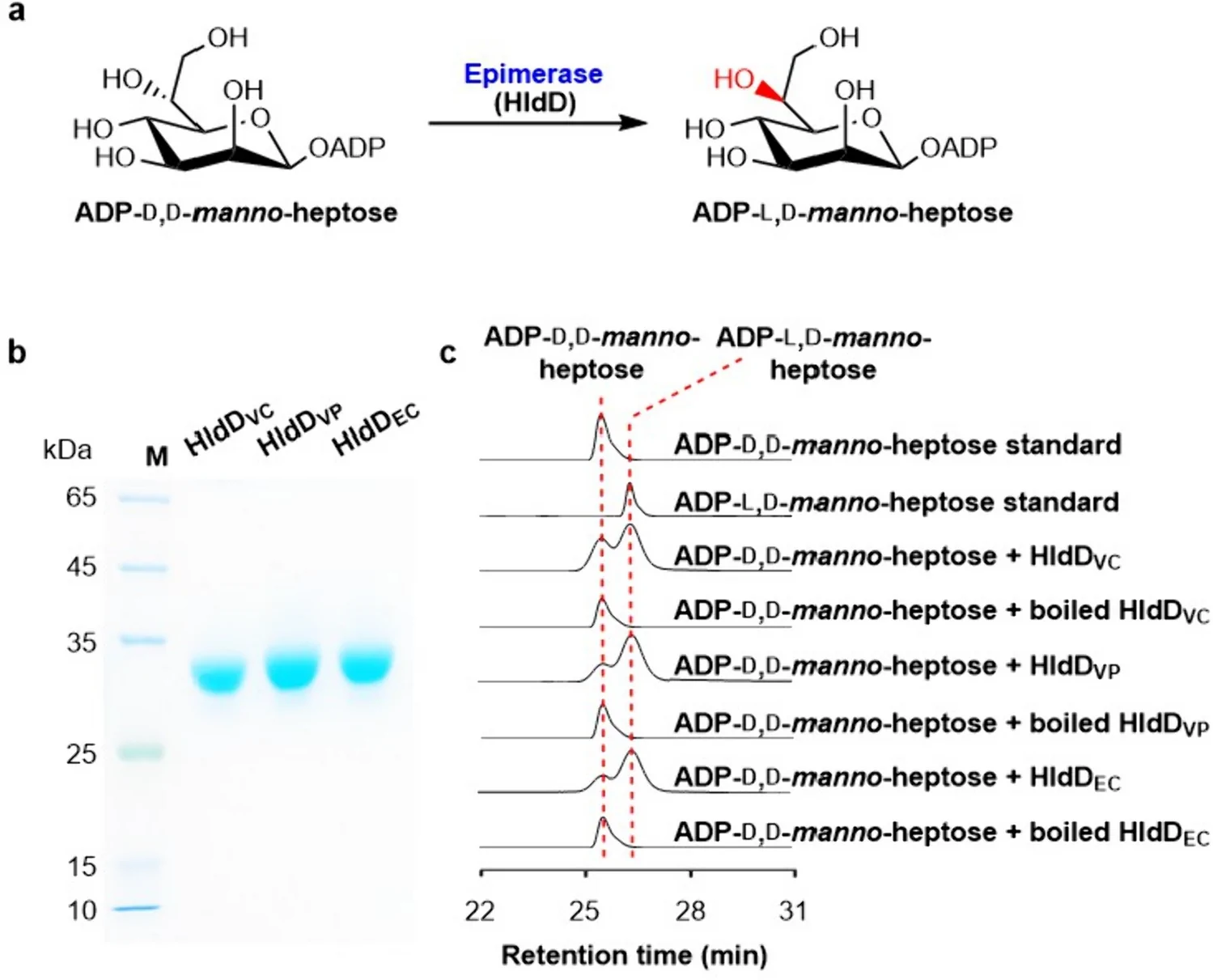
Characterization of the ADP-d,d-*manno*-heptose C-6′′ epimerases from two pathogenic *Vibrio* strains. **a** A proposed reaction to form ADP-l,d-*manno*-heptose catalyzed by ADP-d,d-*manno*-heptose C-6′′ epimerase. **b** SDS-PAGE analysis of *N*-His_6_ tagged ADP-d,d-*manno*-heptose C-6′′ epimerases. Lane M, protein marker. HldD_VC_, HldD_VP_, and HldD_EC_ are from *V. cholerae* O1 2010EL-1786, *V. parahaemolyticus* CGMCC 1.1997, *E. coli* BL21 (DE3), respectively. **c** Representative HPLC profiles of the enzymatic assays of ADP-d,d*-manno*-heptose C-6′′ epimerases. The detection wavelength was set at 254 nm

The biosynthesis of ADP-*β*-d-*manno*-heptoses has been well elucidated in several bacteria. They are derived from d-sedoheptulose 7-phosphate (S7P), an intermediate of the pentose phosphate pathway. In *Escherichia coli*, S7P is converted to d-*glycero*-d-*manno*-heptose 7-phosphate (H7P) by an isomerase GmhA_EC_, and the kinase domain of HldE_EC_, a bifunctional kinase/nucleotidyltransferase, catalyzes the phosphorylation of the anomeric carbon of H7P to generate HBP. After the C-7′′ phosphate group of HBP is removed by a phosphatase GmhB_EC_, H1P is activated by the nucleotidyltransferase domain of HldE_EC_ to generate ADP-d,d-*manno*-heptose (Fig. 1a) (Kneidinger et al. 2002). ADP-d,d-*manno*-heptose can be converted to its C-6″ epimer, ADP-l,d-*manno*-heptose, by an NAD^+^ dependent epimerase HldD_EC_ (Fig. 2a) (Morrison and Tanner 2007). It is noteworthy that, in some bacteria (e.g., *Burkholderia pseudomallei*), the *manno*-heptose kinase and nucleotidyltransferase are not bifunctional proteins like HldE_EC_, but two mono-functional enzymes (Park et al. 2018).

The *Vibrio* genus is ubiquitously found in diverse aquatic and marine habitats. It comprises more than 12 species that can cause human infections. Human diseases caused by pathogenic *Vibrio* species are divided into two types: cholera and non-cholera infections (Baker-Austin et al. 2018). *V. cholerae* is the causative agent of pandemic cholera, an acute watery diarrheal disease spreading via the fecal-oral and person-to-person transmission (Kanungo et al. 2022). Of the non-cholera pathogens, *V. parahaemolyticus* and *Vibrio vulnificus* are the representative species causing vibriosis including gastroenteritis, wound infection, and sepsis (Baker-Austin et al. 2018). Several virulence factors, e.g., enterotoxin, hemolysin, and LPS contribute considerably to the pathogenicity of *Vibrio*, thus being studied intensively (Zhang and Austin 2005). In *V. cholerae*, the LPS inner core is usually composed of at least three l-*glycero*-*α*-d-*manno*-heptoses, which are loaded by inverting glycosyltransferases employing ADP-l,d-*manno*-heptose as a sugar donor (Chatterjee and Chaudhuri 2003). However, little is known about the biosynthesis of ADP-activated *β*-d-*manno*-heptoses in *Vibrio*, let alone the influences of those ADP-*β*-d-*manno*-heptoses on *Vibrio* pathogenesis.

In this work, we analyzed the genomes of two pathogenic *Vibrio* strains, *V. cholerae* O1 2010EL-1786 and *V. parahaemolyticus* CGMCC 1.1997 (ATCC 17802), and found that they possess the same set of ADP-*β*-d*-manno*-heptose biosynthetic enzymes as *E. coli*. Interestingly, though they display high protein sequence similarities with the *E. coli*–derived homologues (>60%), the predicted structural differences between the nucleotidyltransferase domains of the bifunctional HldEs aroused our interests to study their functions elaborately. The catalytic activities of those *Vibrio* enzymes were identified by in comparative investigations along with their isozymes from *E. coli*. Restricted by the availability of H1P, quantitative studies on the *β*-d*-manno*-heptose nucleotidyltransferases are difficult. Here, we show that the bifunctional HldEs from both *Vibrio* and *E. coli* could activate *β*-d-mannose 1-phosphate, but not *α*-d-mannose 1-phosphate, to form ADP-*β*-d-mannose. And the kinetic analyses of their nucleotidyltransferase domains were performed using *β*-d-mannose 1-phosphate, which revealed that they have comparable nucleotidyltransfer efficiencies. Taken together, we characterized the enzymes involved in ADP-*β*-d-*manno*-heptose synthesis in *Vibrio* strains and laid a foundation for further investigations on the influence of heptose metabolism on the virulence of *Vibrio*.

## Materials and methods

### Bacterial strains and plasmids

Bacterial strains and plasmids used in this study are listed in Table S1. *E. coli* JM109 was used for general DNA cloning. *E. coli* BL21 (DE3) Δ*gmhA* Δ*gmhB* Δ*hldE* mutant was used for protein expression (Tang et al. 2022). LB broth and agar were used for the growth of *E. coli* strains at 37 °C. *V. parahaemolyticus* CGMCC 1.1997 was cultured at 37 °C in LB broth and used as the template for cloning the genes involving in ADP-*β*-d*-manno*-heptose biosynthesis from its genome (NCBI RefSeq assembly, GCF_001011015.1). ADP-*β*-d-*manno*-heptose biosynthetic genes from *V. cholerae O1* 2010EL-1786 (NCBI RefSeq assembly, GCF_000166455.1) were obtained by DNA synthesis.

### DNA manipulation and sequence analysis

All PCR primers used in this study were synthesized by GENEWZ Co. (Suzhou, China) and listed in Table S2. DNA synthesis and sequencing were carried out in GENEWIZ Co. (Suzhou, China). PCRs were performed with PrimeSTAR HS DNA polymerase (Takara, Shiga, Japan) or Taq DNA polymerase (TransGene, Beijing, China) according to the manufacturers’ instructions. A BLASTP search was used to predict protein functions (https://blast.ncbi.nlm.nih.gov/Blast.cgi).

### Construction of the protein expression plasmids

To construct the expression plasmids of ADP-*β*-d-*manno*-heptose biosynthetic enzymes from *V. cholerae O1* 2010EL-1786, the 0.6-kb isomerase gene *gmhA*_*VC*_, the 0.6-kb phosphatase gene *gmhB*_*VC*_, the 1.4-kb bifunctional kinase/nucleotidyltransferase gene *hldE*_*VC*_, and the 0.9-kb epimerase gene *hldD*_*VC*_ were synthesized and inserted into the *Nde*I*/Bam*HI sites of pET28a to afford plasmids pET28a-*gmhA*_*VC*_, pET28a-*gmhB*_*VC*_, pET28a-*hldE*_*VC*_, and pET28a-*hldD*_*VC*_. For the construction of plasmids pET28a-*gmhA*_*VP*_, pET28a-*gmhB*_*VP*_, pET28a-*hldE*_*VP*_, and pET28a-*hldD*_*VP*_, the linear pET28a fragment was amplified from the pET28a plasmid and digested with *Dpn*I to eliminate the template plasmid. The 0.6-kb *gmhA*_*VP*_ gene, the 0.6-kb g*mhB*_*VP*_ gene, the 1.4-kb *hldE*_*VP*_ gene, and the 0.9-kb *hldD*_*VP*_ gene were amplified from the genome of *V*. *parahaemolyticus* CGMCC 1.1997 using primer pairs GmhA-VP-1/GmhA-VP-2, GmhB-VP-1/GmhB-VP-2, HldE-VP-1/HldE-VP-2, and HldD-VP-1/HldD-VP-2, respectively. Each of the four fragments contained homologous sequences for ligation independent cloning at its both ends and was ligated with the linear pET28a fragment by one-step clone strategy. The desired plasmids were verified by DNA sequencing.

### Protein expression and purification

All the proteins were expressed in *E. coli* BL21 (DE3) Δ*gmhA*_*EC*_ Δ*gmhB*_*EC*_ Δ*hldE*_*EC*_ mutant. A single transformant of the *E. coli* BL21 Δ*gmhA*_*EC*_ Δ*gmhB*_*EC*_ Δ*hldE*_*EC*_ strain harboring a specific protein expression plasmid was inoculated into LB medium with 50 μg/mL kanamycin and cultured overnight at 37 °C, 220 rpm. Subsequently, the overnight culture was inoculated into LB with 50 μg/mL kanamycin at 1:100 dilution and incubated at 37 °C, 220 rpm until OD_600_ reached 0.6. The expression of the candidate protein was then induced by adding isopropyl *β*-d-thiopyranogalactoside (IPTG) to a final concentration of 0.1 mM and cultured at 16 °C, 180 rpm for 18 h.

Protein purifications were carried out with Ni-NTA affinity column at 4 °C following the manufacturer’s instructions. After harvesting the cell pellets by centrifugation, we resuspended them in binding buffer (20 mM Tris-HCl, 500 mM NaCl, 5 mM imidazole, 5% glycerol, pH 7.9) for sonication. Then, the cell debris was removed by centrifugation and the supernatant was loaded onto Ni-NTA affinity column pre-equilibrated with binding buffer. After being washed with washing buffer (20 mM Tris-HCl, 500 mM NaCl, 60 mM imidazole, 5% glycerol, pH 7.9) and elution buffer (20 mM Tris-HCl, 500 mM NaCl, 500 mM imidazole, 5% glycerol, pH 7.9) sequentially, the desired fractions were combined, desalted with PD-10 columns (GE Healthcare, USA), and concentrated by ultracentrifugation using an Amicon Ultra Centrifugal Filter device (Merck Millipore, USA; molecular mass cutoff of 10 kDa for the bifunctional HldEs and 3 kDa for the other proteins). The purified proteins were stored in 20 mM HEPES buffer (pH 8.0) with 200 mM NaCl and 10% glycerol at −80 °C (Li et al. 2021). Protein concentrations were measured by the Bradford assay (Bradford 1976).

### Assays of the ADP-d,d-*manno*-heptose biosynthetic enzymes

The catalytic activities of ADP-d,d-*manno*-heptose biosynthetic enzymes were studied with the four-step assays using S7P and ATP as substrates. For the characterization of GmhA_VC_, GmhB_VC_, and HldE_VC_ from *V. cholerae* O1 2010EL-1786, the reactions were performed in a 50-μL volume mixture containing 20 mM HEPES buffer (10% glycerol, 200 mM NaCl, pH 8.0), 2 mM MgCl_2_, 2 mM KCl, 2 mM ATP, 0.2 mM S7P, 5 μM GmhA_VC_, 5 μM GmhB_VC_, and 5 μM HldE_VC_ at 30 °C for 2 h. And the well-studied combination of GmhA_EC_, GmhB_EC_, and HldE_EC_ from *E. coli* was performed at the same conditions as a positive control. To check the catalytic activity of GmhA_VC_ or GmhB_VC_ or HldE_VC_, its corresponding isoenzyme in the combination of GmhA_EC_ + GmhB_EC_ + HldE_EC_ was replaced by the one from *V. cholerae* O1 2010EL-1786. The assays of ADP-d,d-*manno*-heptose biosynthetic enzymes from *V. parahaemolyticus* CGMCC 1.1997 and the compensation experiments of their isoenzymes from *E. coli* were performed similarly as the enzymes from *V. cholerae O1* 2010EL-1786. All of the reactions were quenched by mixing vigorously with an equal volume of chloroform. After centrifugation, the chloroform layer was removed and 10 μL of aqueous sample was subjected to HPLC analysis (Tang et al. 2022).

### Enzymatic assays of HldD_VC_, HldD_VP_, and HldD_EC_

The enzymatic reactions of C-6″ epimerases, HldD_VC_, HldD_VP_, and HldD_EC_, were performed at the same conditions and here takes HldD_VC_ as an example. The enzymatic assay of HldD_VC_ was set in a 50-μL volume mixture containing 20 mM HEPES buffer (10% glycerol, 200 mM NaCl, pH 8.0), 2 mM NAD^+^, 0.2 mM ADP-d,d-*manno*-heptose, and 5 μM HldD_VC_ at 30 °C for 1 h. The reaction was quenched by mixing vigorously with an equal volume of chloroform. After centrifugation, the chloroform layer was removed and 10 μL of aqueous sample was subjected to HPLC analysis.

### Enzymatic assays of HldE using *β*-d-mannose 1-phosphate as a substrate

To test whether HldE could use *β*-d-mannose 1-phosphate as a substrate, the enzymatic reaction was carried out in a 50 μL volume mixture containing 20 mM HEPES buffer (10% glycerol, 200 mM NaCl, pH 8.0), 2 mM MgCl_2_, 2 mM ATP, 0.2 mM *β*-d-mannose 1-phosphate (Supplementary Scheme 1 and Fig. S1), and 5 μM HldE_VC_ or HldE_VP_ or HldE_EC_ at 30 °C for 2 h. To test whether *α*-d-mannose 1-phosphate can be taken by HldE, the reactions were performed at the same conditions except that *β*-d-mannose 1-phosphate was replaced by *α*-d-mannose 1-phosphate. All the reactions were quenched by mixing vigorously with an equal volume of chloroform. After centrifugation, the chloroform layer was removed and 10 μL of aqueous sample was subjected to HPLC analysis.

### Preparation of ADP-*β*-d-mannose

ADP-*β*-d-mannose was enzymatically prepared by assays in a 100-μL volume mixture containing 20 mM HEPES buffer (10% glycerol, 200 mM NaCl, pH 8.0), 2 mM MgCl_2_, 2 mM KCl, 5 mM ATP, 1 mM *β*-d-mannose 1-phosphate, and 20 μM HldE_EC_ at 30 °C for 6 h. The reaction mixture was filtrated with an Amicon Ultra 10 kDa centrifugal filter (Merck Millipore, MA, USA) to remove the proteins. ADP-*β*-d-mannose was purified with a Dionex CarboPac™ PA1 BioLC™ Semi-Prep column (9 × 250 mm, Thermo Fisher Scientific, Sunnyvale, CA, USA) on a Shimadzu HPLC system (Shimadzu, Kyoto, Japan) at a detection wavelength of 254 nm. The column was developed at a flow rate of 2.0 mL/min with solvent A (ddH_2_O) and solvent B (1.0 M CH_3_COONH_4_). The percentage of solvent B was changed using the following gradient: 0–2 min, 40%; 2–7 min, 40%–70%; 7–15 min, 70%; 15–16 min, 70%–90%; 16–23 min, 90%; 23–24 min, 90%–40%; and 25–28 min, 40%. The fractions of the desired product were collected, concentrated via lyophilization, and refined on a Sephadex LH20 column (eluted with ddH_2_O) to obtain ADP-*β*-d-mannose.

### Enzymatic assays of ADP-d-*glycero*-*β*-d-*altro*-heptose synthesis

To test whether HldE_VC_ from *V. cholerae* O1 2010EL-1786 can synthesize ADP-d-*glycero*-*β*-d-*altro*-heptose, the enzymatic reactions were performed in a 50-μL volume mixture containing 20 mM HEPES buffer (10% glycerol, 200 mM NaCl, pH 8.0), 2 mM MgCl_2_, 2 mM KCl, 2 mM ATP, 0.2 mM S7P, 5 μM HygP, 5 μM GmhB_VC_, and 5 μM HldE_VC_ at 30 °C for 2 h. The enzymatic assays of HldE_VP_ from *V. parahaemolyticus* CGMCC 1.1997 were performed similarly except that HldE_VC_ and GmhB_VC_ were replaced by HldE_VP_ and GmhB_VP_. The well-studied combination of HygP, GmhB_EC_, and HldE_EC_ was performed as a positive control at the same conditions. All of the reactions were quenched by mixing vigorously with an equal volume of chloroform. After centrifugation, the chloroform layer was removed and 10 μL of aqueous sample was subjected to HPLC analysis.

### Spectroscopic analysis

Analytical HPLC was performed with a Dionex CarboPac™ PA1 BioLC™ column (4 × 250 mm, Thermo Fisher Scientific, USA) on a Shimadzu HPLC system (Shimadzu, Japan). For the detections of ADP-d,d-*manno*-heptose and ADP-*β*-d-mannose, the column was developed with solvent A (ddH_2_O) and solvent B (1.0 M CH_3_COONH_4_) at a flow rate of 1.0 mL/min. The percentage of solvent B was changed using the following gradient: 0–5 min, 15%; 5–30 min, 15%–45%; 30–32 min, 45%–90%; 32–38 min, 90%; 38–40 min, 90%–15%; and 40–45 min, 15%. To detect ADP-l,d-*manno*-heptose, the percentage of solvent B was changed using the following gradient:0–5 min, 24%; 5–30 min, 24%–27%; 30–32 min, 27%–90%; 32–38 min, 90%; 38–40 min, 90%–24%; and 40–45 min, 24%. To detect ADP-d-*glycero*-*β*-d-*altro*-heptose, the percentage of solvent B was changed using the following gradient: 0–5 min, 15%; 5–30 min, 15%–30%; 30–40 min, 30%–45%; 40–42 min, 45%–90%; 42–52 min, 90%; and 52–53 min, 90%–15%; and 53–63 min, 15%. The detection wavelength was set as 254 nm.

HRMS was performed on an Agilent 1260 HPLC/6520 QTOF-MS instrument with an electrospray ionization source. NMR spectra were recorded at room temperature on a Bruker-500 NMR.

### Colorimetric assay

A pyrophosphatase (PPase)-coupled colorimetric assay was developed for monitoring the nucleotidyltransfer activities of HldE_VC_, HldE_VP_, and HldE_EC_. Briefly, the enzymatic assay was carried out in a 50-μL volume mixture in 96-well microtiter plate using ATP (0.2 mM) and *β*-d-mannose 1-phosphate (0.1 mM) as substrates. The PPase assays revealed that 0.1 U PPase could effectively catalyze the hydrolysis of 0.2 mM PPi at a wide temperature range (from 15 to 45 °C). Therefore, HldE was added together with 0.1 U PPase and the reaction mixture was incubated at the assay temperature for HldE. After 30 min, the reactions were terminated by adding the premixed malachite green and ammonium molybdate agent from the Malachite Green Phosphate Detection Kit (CST, MA, USA) at room temperature for 15 min and monitored by a microplate reader (Synergy H4, BioTek, USA) at 630 nm (Sha et al. 2012). The reactions with boiled HldEs were carried out as negative controls to adjust the interference by substrates and buffer components.

### Kinetic studies

Using the developed colorimetric assay, the reaction conditions of HldE_VC_, HldE_VP_, and HldE_EC_ were optimized. The optimal pH was determined by performing the enzyme reaction in 20 mM different buffers (citrate-sodium citrate buffer (pH 6.5), HEPES-NaOH (pH 7.0, 7.5, 8.0), Tris-HCl buffer (pH 8.5, 9.0), glycine-NaOH buffer (pH 9.5, 10.0)) at 30 °C. The optimal temperatures of the three enzymes were determined by incubating the reactions at 15 to 45 °C at their optimal pHs. The initial velocities were evaluated by performing the reactions at a different incubation time under the optimal pH and temperature.

The *K*_m_ and *k*_cat_ values of HldE_VC_, HldE_VP_, and HldE_EC_ against *β*-d-mannose 1-phosphate and ATP were determined under their optimal pH and temperature. The *K*_*m*_ values of the three enzymes against *β*-d-mannose 1-phosphate were obtained by performing the reactions with different concentrations of *β*-d-mannose 1-phosphate (5, 10, 15, 20, 25, 30, 35, 40, 45, 50, 75, 100, 125, and 150 μM) and a saturation concentration of ATP (2 mM). The *K*_*m*_ values of the three enzymes against ATP were obtained by performing the reactions with different concentrations of ATP (20, 35, 50, 75, 100, 150, 200, 250, 300, 500, 750, and 1000 μM) and a saturation concentration of *β*-d-mannose 1-phosphate (400 μM). Each parameter was measured in triplicate and the data were analyzed using Origin 2021.

## Results

### Characterization of the *Vibrio* enzymes responsible for ADP-d,d-*manno*-heptose biosynthesis

*V. cholerae* O1 2010EL-1786 is a causative agent of life-threating cholera isolated from a stool sample of one cholera patient (Reimer et al. 2011); *V. parahaemolyticus* CGMCC 1.1997 is a gastroenteritis causative strain from a patient suffering with Shirasu food poisoning (Daniel et al. 1999). *In silico* analysis of their genome sequences (NCBI RefSeq assemblies: GCF_000166455.1 (*V. cholerae* O1 2010EL-1786) and GCF_001011015.1 (*V. parahaemolyticus* CGMCC 1.1997)) revealed that both of the two strains contain the necessary genes responsible for ADP-*β*-d-*manno*-heptose biosynthesis. Sequence alignments revealed that the ADP-*β*-d-*manno*-heptose biosynthetic enzymes from the two *Vibrio* strains display more than 60% amino acid sequence identities with their homologues from *E. coli* (Table S3). AlphaFold prediction and subsequent structure analysis revealed that the overall structures of the isomerases, phosphatases, and ADP-l,d-*manno*-heptose C-6″ epimerases from both *Vibrio* strains are quite similar to their homologues from *E. coli* (Figs. S2 and S3), while prominent conformation differences that deviate severely from HldE_EC_ were observed in both of the two HldEs from *Vibrio*, especially the nucleotidyltransferase domain of HldE_VP_ (Fig. 1c), which aroused our interests to study their functions elaborately. We cloned the genes encoding *β*-d*-manno*-heptose isomerase (*gmhA*_*VP*_), phosphatase (*gmhB*_*VP*_), and bifunctional kinase/nucleotidyltransferase (*hldE*_*VP*_) from *V. parahaemolyticus* CGMCC 1.1997. The genes encoding the same set of isozymes from *V. cholerae* O1 2010EL-1786, *gmhA*_*VC*_, *gmhB*_*VC*_, and *hldE*_*VC*_, were codon optimized and synthesized. The six genes were expressed as *N*-His_6_ tagged proteins using a “clean” chassis strain *E. coli* BL21 (DE3) Δ*gmhA* Δ*gmhB* Δ*hldE* (Tang et al. 2022), in which the native *β*-d*-manno*-heptose biosynthetic genes were knocked out (Fig. 1c).

The catalytic activities of the enzymes responsible for ADP-d,d*-manno*-heptose synthesis in *V. cholerae* O1 2010EL-1786 were investigated by incubating GmhA_VC_, GmhB_VC_, and HldE_VC_ with S7P and ATP. A product shared the same retention time with ADP-d,d*-manno*-heptose was generated and was further identified by HPLC co-injection with the authentic standard of ADP-d,d*-manno*-heptose. We also tested the hybrid assays by replacing the well-characterized isomerase, phosphatase, or kinase/nucleotidyltransferase in the combination of GmhA_EC_ + GmhB_EC_ + HldE_EC_, which can synthesize ADP-d,d*-manno*-heptose efficiently, with the corresponding isozyme from *V. cholerae* O1 2010EL-1786, and all the combinations converted S7P to ADP-d,d*-manno*-heptose with comparable efficiencies (Fig. 1d).

When the heptose synthetic enzymes from *V. parahaemolyticus* CGMCC 1.1997 were incubated with S7P and ATP, ADP-d,d*-manno*-heptose was also generated in the GmhA_VP_ + GmhB_VP_ + HldE_VP_ combination, but not in the control reaction with boiled HldE_VP_. And the enzymes from *V. parahaemolyticus* CGMCC 1.1997 could also functionally replace their isozymes in the GmhA_EC_ + GmhB_EC_ + HldE_EC_ combination (Fig. 1d). HPLC analyses showed that about 72 μM, 64 μM, and 80 μM of S7P (200 μM) were converted to ADP-d,d*-manno*-heptose by HldE_VC_, HldE_VP_, and HldE_EC_ together with its cognate GmhA and GmhB, respectively, at 30 °C, pH 8.0 (20 mM HEPES) for 2 h. The comparable production of ADP-d,d*-manno*-heptose could be explained by docking analysis of ATP in the binding pockets of the nucleotidyltransferase domains of HldEs, which showed that despite the differences in tertiary structures of the HldEs, they may adopt similar “horseshoe” binding model of ATP to shape ADP-d,d*-manno*-heptose effectively (Fig. S4). Collectively, these results confirmed that GmhA_VC_ and GmhA_VP_ are S7P isomerases, GmhB_VC_ and GmhB_VP_ are HBP phosphatases, and HldE_VC_ and HldE_VP_ are bifunctional proteins with H7P kinase and H1P nucleotidyltransferase activities.

### Conversion of ADP-d,d-*manno*-heptose to ADP-l,d-*manno*-heptose by a C-6″ epimerase

To our knowledge, three l,d-*manno*-heptoses are contained in the LPS inner core of *V. cholerae* O1 strains as in *E. coli*, while the LPS structure of *V. parahaemolyticus* CGMCC 1.1997 remains unknown (Chatterjee and Chaudhuri 2003). In *E. coli*, ADP-d,d-*manno*-heptose is converted to ADP-l,d-*manno*-heptose by an NAD^+^-dependent C-6″ epimerase HldD_EC_ (Fig. 2a). Bioinformatic analysis revealed that both of the two *Vibrio* genomes contain an isoenzyme HldD_EC_, HldD_VC_ (WP_000587795.1), and HldD_VP_ (WP_015296139.1). Therefore, we cloned the epimerase encoding gene *hldD*_*VP*_ from *V. parahaemolyticus* CGMCC 1.1997, and the *hldD*_*VC*_ gene from *V. cholerae* O1 2010EL-1786 was obtained by DNA synthesis after codon optimization. The two epimerases were then expressed as *N*-His_6_ tagged proteins in *E. coli* BL21 (DE3) Δ*gmhA* Δ*gmhB* Δ*hldE* (Fig. 2b).

To verify the function of HldD_VC_, it was incubated with ADP-d,d*-manno*-heptose and NAD^+^. HPLC analysis showed that ADP-d,d*-manno*-heptose was converted to a compound having the same retention time as the authentic standard of ADP-l,d-*manno*-heptose, and it was further verified by HRMS analysis (*m*/*z* 618.0847 for [M-H]^−^,C_17_H_27_N_5_O_16_P_2_, *cacld* 618.0855) (Fig. S5). HldD_VP_ could also convert ADP-d,d*-manno*-heptose to ADP-l,d*-manno*-heptose under the same assay conditions. Both of the two ADP-d,d*-manno*-heptose C-6″ epimerase from *Vibrio* species displayed comparable catalytic efficiencies with the *E. coli* epimerase HldD_EC_, which was used as a positive control (Fig. 2c). Taken together, we showed that both *V. cholerae* O1 2010EL-1786 and *V. parahaemolyticus* CGMCC 1.1997 possess the complete set of *β*-d*-manno*-heptose biosynthetic enzymes for synthesizing ADP-l,d*-manno*-heptose from S7P, implying that l,d*-manno*-heptose is widely distributed in the LPSs of different *Vibrio* strains.

### Synthesis of ADP-*β*-d-mannose by the nucleotidyltransferase domain of HldE

**Fig. 3:**
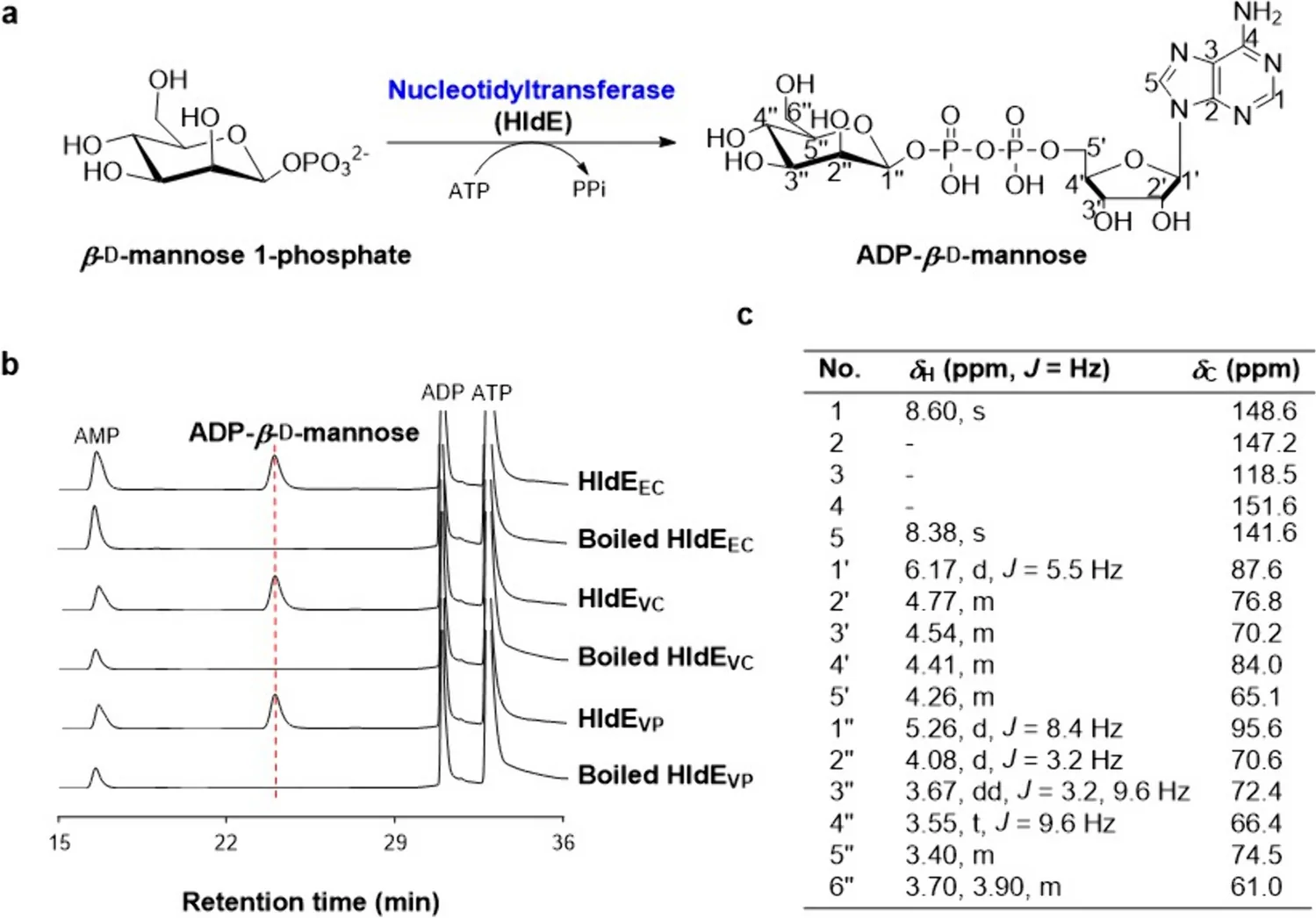
Conversion of *β*-d-mannose 1-phosphate to ADP-*β*-d-mannose. **a** A proposed reaction to form ADP-*β*-d-mannose. **b** HPLC profiles of the enzymatic assays catalyzed by the nucleotidyltransferase domains of HldEs using *β*-d-mannose 1-phosphate and ATP as substrates. The detection wavelength was set as 254 nm. **c** ^1^H NMR (500 MHz) and ^13^C NMR (125 MHz) data for ADP-*β*-d-mannose in D_2_O

A study on HldE_EC_ suggested that it can convert mannose to ADP-*β*-d-mannose in the presence of ATP, indicating that the kinase domain of HldE_EC_ is able to add a phosphate group onto the anomeric carbon of mannose to form *β*-d-mannose 1-phosphate, which is then converted to ADP-*β*-d-mannose by the nucleotidyltransferase domain of HldE_EC_ (Morrison and Tanner 2007). To verify the substrate promiscuity of the nucleotidyltransferase domain of HldE_EC_, we chemically synthesized *β*-d-mannose 1-phosphate as described (Supplementary Scheme 1 and Fig. S1). When HldE_EC_ was incubated with ATP and the mimic sugar substrate, *β*-d-mannose 1-phosphate, or *α*-d-mannose 1-phosphate (commercially available), a new peak was observed only in the assay of *β*-d-mannose 1-phosphate, while not in the assay using *α*-d-mannose 1-phosphate or the negative control using boiled HldE_EC_ (Fig. 3b; Fig. S6). Subsequently, the product was prepared by a large-scale enzymatic synthesis and confirmed to be ADP-*β*-d-mannose by careful analyses of its high-resolution mass spectrometry and nuclear magnetic resonance data (Fig. 3c). The *β*-configuration of the anomeric carbon of mannose was assigned by the NOE correlations of H-1″ with H-3″ and H-5″ (Fig. S7). We also tested the catalytic activities of HldE_VC_ and HldE_VP_ toward *β*-d-mannose 1-phosphate (200 μM) and *α*-d-mannose 1-phosphate. Both of the two enzymes could only take *β*-d-mannose 1-phosphate, activate it into ADP-*β*-d-mannose as HldE_EC_, and comparable yields of ADP-*β*-d-mannose (76 μM for HldE_VC_, 68 μM for HldE_VP_, and 76 μM for HldE_EC_) were detected at 30 °C, pH 8.0 (20 mM HEPES), 2 h (Fig. 3a, b; Fig. S6). The results suggested that the nucleotidyltransferase domain of HldE possesses a certain level of promiscuity toward the size of the sugar substrates, but it has a stringent specificity on the anomeric configuration of sugar 1-phosphate.

### Kinetics of the nucleotidyltransferase domains of HldE_VC_, HldE_VP_, and HldE_EC_

As aforementioned, ADP-*β*-d-*manno*-heptoses are important components of LPS. However, due to the difficulties in preparing H1P, the catalytic efficiency of the nucleotidyltransferase domain of HldE, which is responsible for activating H1P into ADP-d,d*-manno*-heptose, has not been investigated kinetically yet. If the real substrate of a nucleotidyltransferase is unavailable, its mimic substrate can be used instead for kinetic studies (Kim et al. 2020; Kim et al. 2021). With *β*-d-mannose 1-phosphate in hand, we collected the kinetic parameters of HldE using it and ATP as substrates.

**Fig. 4:**
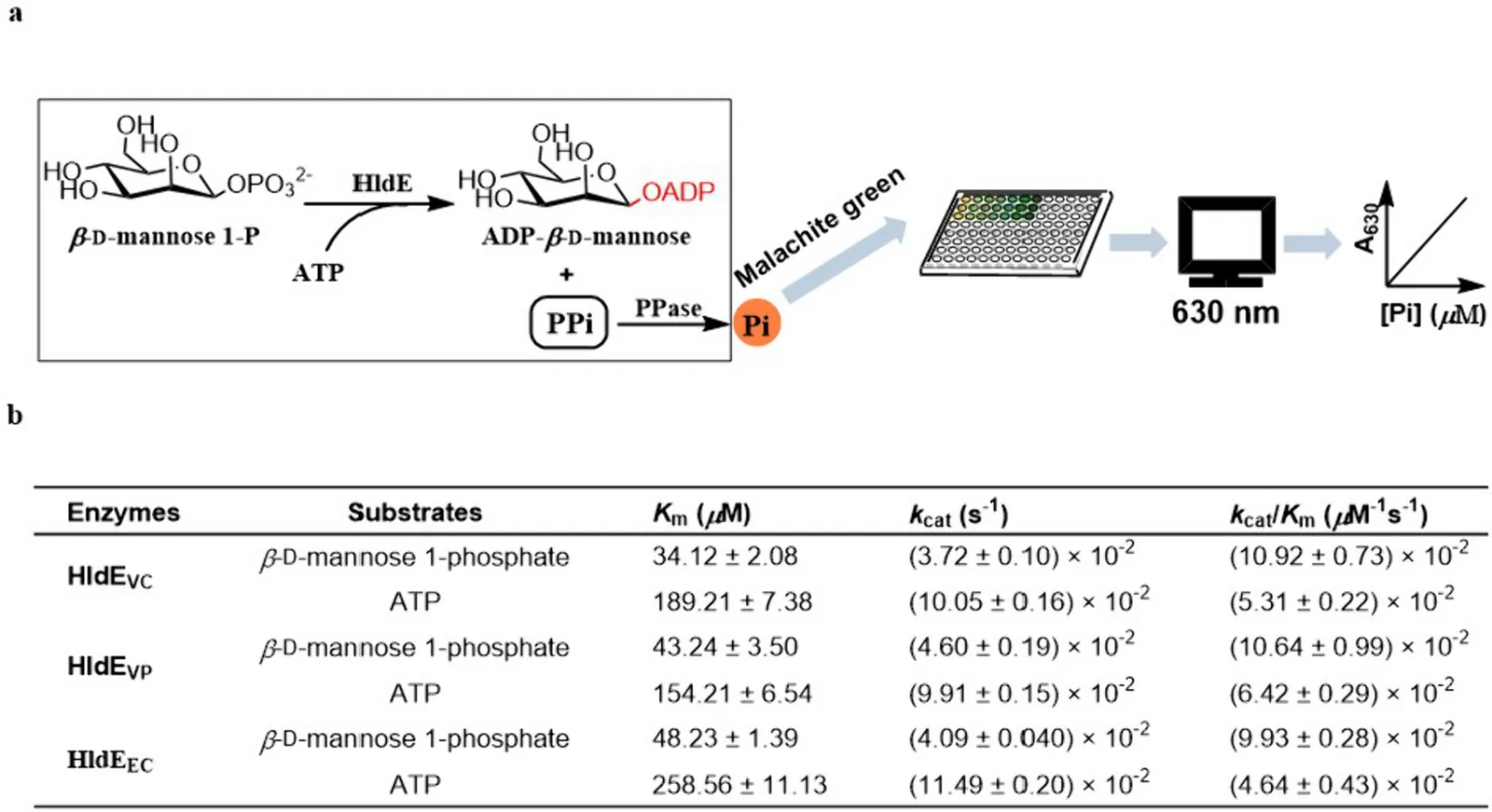
Determination of the kinetics of the nucleotidyltransferase domains of HldEs. **a** A scheme of the colorimetric assay for detecting the formed PPi group that is generated by the nucleotidyltransfer reaction. **b** The kinetic parameters of the nucleotidyltransferase domains of HldEs against different substrates

The nucleotidyltransferase domain of HldE cleaves an ATP into an AMP and one molecule of pyrophosphoric acid (PPi). The AMP is employed to activate *β*-d-pyranose 1-phosphate into ADP-*β*-d-pyranose, and the PPi can be hydrolyzed into inorganic phosphate (Pi) by a PPase and measured conveniently by a colorimetric assay as shown in Fig. 4a. Pi reacts with the premixed malachite green and ammonium molybdate agent under acidic condition to generate a malachite green complex, and the signal can be easily read at 630 nm. We developed a stable detection process by controlling the reaction and the detection conditions to minimize the background influence. Then, the reaction conditions of HldE_VC_ and HldE_VP_ as well as HldE_EC_ were optimized by measuring the Pi generation during the process. It was showed that the three enzymes reached their maximal activities at 30 °C, and the optimum pHs of HldE_VC_, HldE_VP_, and HldE_EC_ were 8.5, 8.0, and 7.5, respectively (Figs. S8 and S9). Subsequently, steady-state kinetic studies were carried out under the optimized conditions. The *K*_m_ and *k*_cat_ values against both substrates, *β*-d-mannose 1-phosphate and ATP, were collected and summarized in Fig. 4b. HldE_VC_ and HldE_VP_ displayed comparable *K*_m_ and *k*_cat_ values. HldE_EC_ from *E. coli* exhibited slightly higher affinities to *β*-d-mannose 1-phosphate and ATP substrates than HldEs from *Vibrio* species, while it had comparable turnover numbers (Fig. 4b; Fig. S10).

## Discussion

*Vibrio* genus contains several important pathogenic species (e.g., *V. cholerae* and *V. parahaemolyticus*) that can cause human diseases. Many people around the world are suffering waterborne and foodborne vibriosis with symptomatic entities such as watery diarrhea, stomach cramping, nausea, vomiting, fever, and chills (Dutta et al. 2021). ADP-*β*-d-*manno*-heptoses participate in the assembly of LPS, a virulence factor of *Vibrio* infections (Qadri et al. 2003; Chatterjee and Chaudhuri 2003), and are also powerful agonists that could trigger NF-*κ*B-mediated innate immune responses (Janeway Jr. and Medzhitov 2002; Zhou et al. 2018). However, little is known about the formation mechanism of ADP-*β*-d-*manno*-heptoses in *Vibrio* species or the influences of those ADP-sugars on their pathogenesis. In this work, we characterized the *manno*-heptose biosynthetic enzymes from two *Vibrio* strains and proposed that *Vibrio* adopts the same biosynthetic pathway as *E. coli* to synthesize ADP-d,d-*manno*-heptose and then ADP-l,d-*manno*-heptose using S7P as a precursor. In this process, S7P goes through a four-step reaction relay including isomerization, phosphorylation at C-1″, dephosphorylation at C-7″, and nucleotide activation to form ADP-d,d-*manno*-heptose. ADP-d,d-*manno*-heptose is further epimerized at C-6″ to generate ADP-l,d-*manno*-heptose, which is then loaded to lipid A to assemble the inner core of LPS. The results showed that *Vibrio* strains synthesize ADP-*β*-d-*manno*-heptoses as most of the other Gram-negative bacteria. Characterization of the enzymes involved in *manno*-heptose biosynthesis paves the way for further investigations on the influences of ADP-*β*-d-*manno*-heptoses on *Vibrio* pathogenesis.

Conversion of H1P to ADP-d,d-*manno*-heptose by nucleotidyltransferase is a key step of *β*-d-*manno*-heptose metabolism. It activates the *β*-d-*manno*-heptose into its ADP form, which can be further modified by the following C-6″ epimerase and facilitate the addition of *β*-d-*manno*-heptose to lipid A (Whitfield and Trent 2014). To date, a number of different H1P nucleotidyltransferases have been studied enzymatically (Kneidinger et al. 2002; Park et al. 2018; Tang et al. 2022). However, none of them, even the well-characterized HldE_EC_ from *E. coli*, has been investigated kinetically, mainly due to the difficulties in obtaining the sugar substrate, H1P. We chemically synthesized a mimic substrate, *β*-d-mannose 1-phosphate, and verified that all of the HldEs from *E. coli* and *Vibrio* strains could take it and catalyze the conversion of *β*-d-mannose 1-phosphate to ADP-*β*-d-mannose with considerable efficiencies. Thus, kinetic analyses of the three HldEs were performed using the synthesized *β*-d-mannose 1-phosphate as the sugar donor and ATP as the acceptor. The results showed that the two *Vibrio* enzymes, HldE_VC_ and HldE_VP_, exhibited comparable nucleotidyltransfer efficiencies, while HldE_EC_ from *E. coli* outperformed a little bit on affinities of both substrates. Actually, if the real substrate of a nucleotidyltransferase is unavailable, its mimic substrate can be used instead for kinetic studies (Kim et al. 2020; Kim et al. 2021). While this approach may not capture the natural properties of the enzyme, it can still provide us valuable information and enhance our understanding to the targeted enzyme. In addition, our previous works revealed that HldE_EC_ is able to tolerate the 3-epimer of H1P and activate d-*glycero*-*β*-d-*altro*-heptose 1-phosphate into its ADP form (Tang et al. 2018) and the *Vibrio* strain–derived HldE_VC_ and HldE_VP_ also possess similar abilities to synthesize ADP-d-*glycero*-*β*-d-*altro*-heptose (Fig. S11). Taken collectively, HldEs exhibit a certain level of sugar substrate flexibilities and can take not only different *β*-d-heptose 1-phosphate but also *β*-d-mannose 1-phosphate (Adekoya et al. 2018). They may be developed as potent catalysts for the synthesis of various ADP-*β*-d-sugars that are not easily synthesized by chemical methods.

In summary, we characterized the biosynthetic enzymes of ADP-*β*-d-*manno*-heptoses from two pathogenic *Vibrio* strains, which suggested that *Vibrio* strains adopt the same biosynthetic pathway as *E. coli* in synthesizing ADP-*β*-d-*manno*-heptoses. Moreover, we showed that the two HldEs from *Vibrio* could activate *β*-d-mannose 1-phosphate to its ADP form as well as HldE_EC_ from *E. coli* and studied the kinetics of the nucleotidyltransferase domains of these three HldEs using this mimic substrate. All of our works enhance our understanding of ADP-*β*-d-*manno*-heptose biosynthesis in *Vibrio* strains and laid a foundation for the following studies on heptose metabolism in *Vibrio* and its influences on *Vibrio* pathogenesis.

## Supplementary information

ESM 1 (PDF 1832 kb)

## Data Availability

The original GenBank accession numbers of *gmhA*_*VC*_, *hldE*_*VC*_, *gmhB*_*VC*_, and *hldD*_*VC*_ from *V. cholerae* O1 2010EL-1786 are WP_000284054.1, WP_000805769.1, WP_001108094.1, and WP_000587795.1, respectively. The nucleotide sequence of codon-optimized *gmhA*_*VC*_ (accession number: OR656557), *hldE*_*VC*_ (accession number: OR656559), *gmhB*_*VC*_ (accession number: OR656558), and *hldD*_*VC*_ (accession number: OR656560) genes for *E. coli* is uploaded to the NCBI database, and the data that support the findings of this study are available from the corresponding author upon reasonable request.
