# Supplementary material for: Characterization of the ADP-β-d-manno-heptose biosynthetic enzymes from two pathogenic Vibrio strains
Source: Appl Microbiol Biotechnol. 2024 Mar 18;108:267. doi: 10.1007/s00253-024-13108-3 (PMC10948575; doi:10.1007/s00253-024-13108-3)
Supplement: Supplementary file 1 [file 253_2024_13108_MOESM1_ESM.pdf]

Characterization of the ADP-*β*-D-*manno*-heptose biosynthetic enzymes from two pathogenic *Vibrio* strains

Zhaoxiang Shi[1,†] · Yue Tang[2,†] · Zhenyi Wang[3] · Min Wang[4] · Zijian Zhong[2,5] · Jingming Jia[1] · Yihua Chen[2,5,*]

[1]School of Traditional Chinese Materia Medica, Shenyang Pharmaceutical University, Shenyang, 117004, China.

[2]State Key Laboratory of Microbial Resources, Institute of Microbiology, Chinese Academy of Sciences, Beijing, 100101, China.

[3]RDFZ Xishan School, Beijing, 100193, China.

[4]School of Biotechnology and Health Sciences, Wuyi University, Jiangmen, 529020, China.

[5]University of Chinese Academy of Sciences, Beijing, 100049, China.

*Corresponding author: Email: chenyihua@im.ac.cn (Y.C.)

†These authors contributed equally to this work.

Chemical synthesis of *β*-D-mannose 1-phosphate

In brief, peracetylated D-mannose (**1**) was obtained by acetylation of D-mannose in the presence of 4-dimethylaminopyridine (DMAP), pyridine, and acetic anhydride (AC_2O). And, selective deacetylation at C-1 position of **1** by treatment with *N*,*N*-diisopropylethylamine (DIPEA) and ammonium acetate yielded **2**, which was phosphorylated using diphenyl chlorophosphate and DMAP in dichloromethane (DCM) to furnish **3**. The deprotection of phenyl groups of **3** was achieved using catalyst PtO_2 under H_2 and the resultant product was further subjected to deacetylation in the presence of MeOH, Et_3N, and H_2O to afford the desired *β*-D-mannose 1-phosphate (Sabesan and Neira 1992).

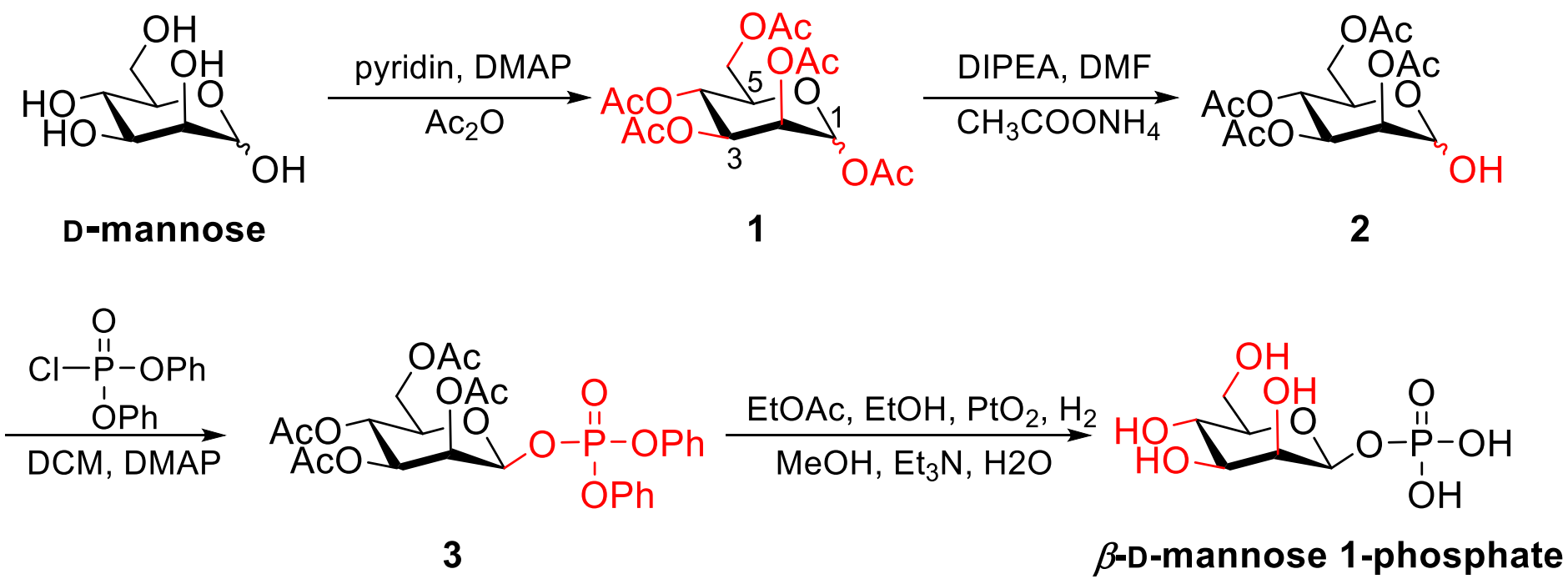

Scheme 1 Synthesis of *β*-D-mannose 1-phosphate.

Synthesis of compound 1

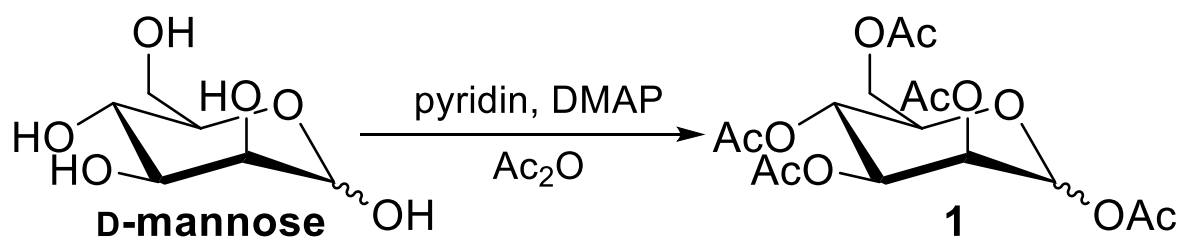

Acetic anhydride (2.68 mL, 28.0 mmol) and 4-dimethylaminopyridine (DMAP) (0.14 g, 1.14 mmol) were added to a solution of D-mannose (0.50 g, 2.80 mmol) in pyridine (4.58 mL, 57.0 mmol) at 0 °C. The resulting mixture was stirred at room temperature for 3 hours. The product was diluted by 50 mL dichloromethane, washed with 2 × 50 mL sat. aq. $NaHCO_3$, and 50 mL brine, sequentially. After dried over sodium sulfate, the solution was filtered and concentrated. The residue was purified by silica-gel column chromatography to obtain compound **1** (0.90 g, 83%).

Synthesis of compound 2

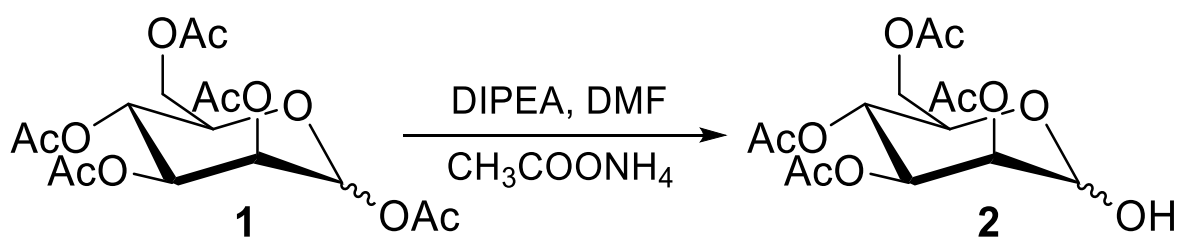

A solution of **1** (0.2 g, 0.51 mmol) in a mixture of dry DMF (10 mL) and *N*,*N*-diisopropylethylamine (0.42 mL, 2.50 mmol) with ammonium acetate (0.16 g, 2.04 mmol), which was prewashed with dry ether and dried over high vacuum pump overnight at 40 °C, was stirred overnight. After removing the insoluble crystals of ammonium acetate by filtration, the reaction mixture was diluted with 50 mL CH_2Cl_2, and then washed with 2 × 50 mL 1M HCl, 2 × 50 mL sat. aq. $NaHCO_3$, 50 mL brine sequentially, subsequently. It was then dried over $MgSO_4$ and concentrated under reduced pressure. Finally, the residue was purified by silica-gel column chromatography to give compound **2** (0.12 g, 68%).

Synthesis of compound 3

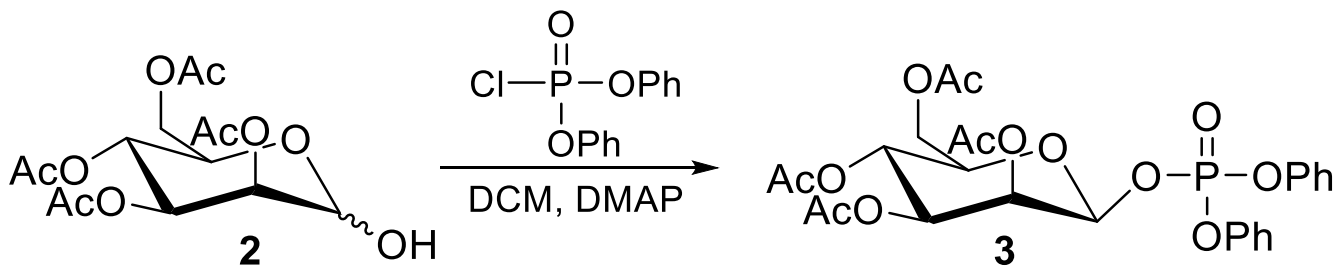

To a solution of compound **2** (0.1 g) in CH_2Cl_2 (3 mL) containing DMAP (0.08 g), diphenyl chlorophosphate (0.14 mL) in CH_2Cl_2 (1 mL) was added during 1 h at room temperature. After 2 h, the products of the reaction were purified by silica-gel column chromatography to afford compound **3** (0.12 g, 70%).

Synthesis of *β*-D-mannose 1-phosphate.

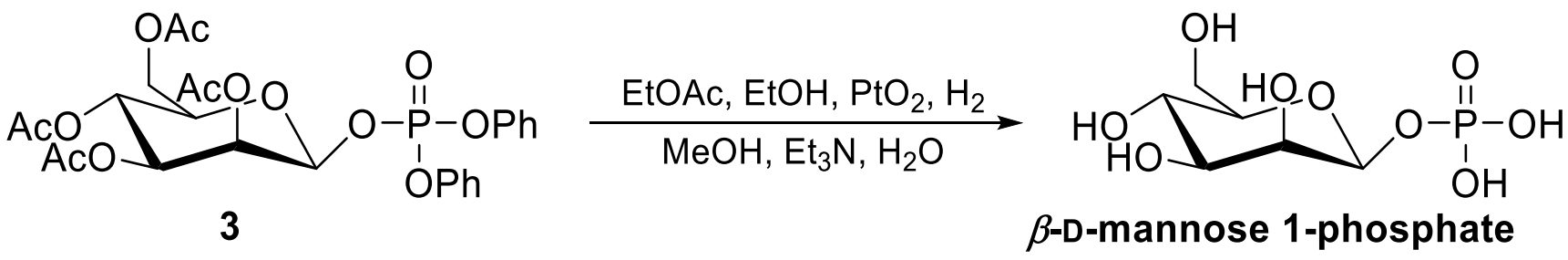

A solution of compound **3** (0.1 g) in 2 mL EtOAc-EtOH (1:1) was hydrogenated in the presence of PtO_2 catalyst (2 mg) for 16 h. After removing the catalyst by filtration, the solution was neutralized with Et_3N and evaporated to give a syrup, which was dissolved in 4 mL MeOH-Et_3N-H_2O (2:1:1) and kept for 5 days at 0 °C. After evaporation, the dried residue was re-dissolved in water, lyophilized, and purified by sephadex LH-20 to afford *β*-D-mannose 1-phosphate as a colorless solid (0.03 g, 67% yield).

***β*-D-mannose 1-phosphate**

^{1}H NMR (500 MHz, D_2O): *δ* 5.26 (dd, *J* = 1.6 and 8.0 Hz, 1H, H-1), 3.85 (m, 1H, H-2), 3.80 (m, 1H, H-4), 3.78 (m, 1H, H-3), 3.75, 3.64 (m, 2H, H-6), 3.53 (m, 1H, H-5).

HRMS (*m*/*z*): calculated for $C_6H_{14}O_9P^+$ $[M+H]^+$: 261.0370, found: 261.0380.

HRMS, ^{1}H NMR, and COSY spectra, see Fig. S1.

ADP-*β*-D-mannose

^{1}H NMR (500 MHz, D_2O): *δ* 8.60 (s, 1H, H-1), 8.38 (s, 1H, H-5), 6.17 (d, *J* = 5.5 Hz, 1H, H-1'), 5.26 (d, *J* = 8.4 Hz,

1H, H-1''), 4.77 (m, 1H, H-2'), 4.54 (m, 1H, H-3'), 4.41 (m, 1H, H-4'), 4.26 (m, 2H, H-5'), 4.08 (d, J = 3.2 Hz, 1H, H-2''), 3.90, 3.70 (m, 2H, H-6'') 3.67 (dd, J = 3.2 and 9.6 Hz, 1H, H-3''), 3.55 (t, J = 9.6 Hz, 1H, H-4''), 3.40 (m, 1H, H-5'').

^{13}C NMR (125 MHz, D_2O): δ 151.6, 148.6, 147.2, 141.6, 118.5, 95.6, 87.6, 84.0, 76.8, 74.5, 72.4, 70.6, 70.2, 66.4, 65.1, 61.0.

HRMS (m/z): calculated for $C_{16}H_{24}N_5O_{15}P_2^-$ [M-H]$^-$: 588.0750, found: 588.0754.

HRMS, ^{1}H NMR and ^{13}C NMR, COSY, and NOE spectra, see Fig. S2.

Table S1 Bacterial strains and plasmids used in this study.

Strains or plasmids	Characteristics (protein ID)	Reference or sources
E. coli		
JM109	General cloning host	Invitrogen
BL21 (DE3) Δ*gmhA*$_{EC}$ Δ*gmhB*$_{EC}$ Δ*hldE*$_{EC}$	Host strain for protein expression	(Tang et al. 2022)
V. parahaemolyticus CGMCC 1.1997	Template for cloning the encoding genes of ADP-*β*-D-*manno*-heptose biosynthesis	This study
**Plasmids**		
pET28a	Kan^r, protein expression vector	Novagen
pET28a/GmhA$_{VC}$	Kan^r, for the expression of *V. cholerae O1* 2010EL-1786 derived GmhA$_{VC}$ (OR656557)	This study
pET28a/GmhB$_{VC}$	Kan^r, for the expression of *V. cholerae O1* 2010EL-1786 derived GmhB$_{VC}$ (OR656558)	This study
pET28a/HldE$_{VC}$	Kan^r, for the expression of *V. cholerae O1* 2010EL-1786 derived HldE$_{VC}$ (OR656559)	This study
pET28a/HldD$_{VC}$	Kan^r, for the expression of *V. cholerae O1* 2010EL-1786 derived HldD$_{VC}$ (OR656560)	This study
pET28a/GmhA$_{VP}$	Kan^r, for the expression of *V. parahaemolyticus* CGMCC 1.1997 derived GmhA$_{VP}$ (WP_005457680.1)	This study
pET28a/GmhB$_{VP}$	Kan^r, for the expression of *V. parahaemolyticus* CGMCC 1.1997 derived GmhB$_{VP}$ (WP_005459163.1)	This study
pET28a/HldE$_{VP}$	Kan^r, for the expression of *V. parahaemolyticus* CGMCC 1.1997 derived HldE$_{VP}$ (WP_005497052.1)	This study
pET28a/HldD$_{VP}$	Kan^r, for the expression of *V. parahaemolyticus* CGMCC 1.1997 derived HldD$_{VP}$ (WP_015296139.1)	This study
pET28a/GmhA$_{EC}$	Kan^r, for the expression of *E. coli* BL21(DE3) derived GmhA$_{EC}$ (WP_000284050.1)	(Tang et al. 2018)
pET28a/GmhB$_{EC}$	Kan^r, for the expression of *E. coli* BL21(DE3) derived GmhB$_{EC}$ (WP_001140174.1)	(Tang et al. 2018)
pET28a/HldE$_{EC}$	Kan^r, for the expression of *E. coli* BL21(DE3) derived HldE$_{EC}$ (WP_000869178.1)	(Tang et al. 2018)
pET28a/HldD$_{EC}$	Kan^r, for the expression of *E. coli* BL21(DE3) derived HldD$_{EC}$ (WP_000587764.1)	(Tang et al. 2018)
pET28a/HygP	Kan^r, for the expression of *E. coli* BL21(DE3) derived HygP (WP_063817660.1)	(Tang et al. 2018)

Table S2 Primers used in this study.

Primers	Sequence (5′ to 3′)	Uses
pET28a-1	AACTCGAGCACCACCACCACC	Confirmation of pET28a derivatives
pET28a-2	GGATCCGCGACCCATTTGCT	
GmhA-VP-1	AGCAAATGGGTCGCGGATCCATGTACCAAGATCTGATTAG	For the expression of $GmhA_{VP}$ from *V. parahaemolyticus* CGMCC 1.1997
GmhA-VP-2	GGTGGTGGTGGTGCTCGAGTTTATTCCATCTCTTTTTCAAT	
GmhB-VP-1	AGCAAATGGGTCGCGGATCCTTGGCAAAACCTGCTGTTTTTAT	For the expression of $GmhB_{VP}$ from *V. parahaemolyticus* CGMCC 1.1997
GmhB-VP-2	GGTGGTGGTGGTGCTCGAGTTTATTTTTGTAAATACGCTG	
HldE-VP-1	AGCAAATGGGTCGCGGATCCATGAAACCAATTCTACCTGAT	For the expression of $HldE_{VP}$ from *V. parahaemolyticus* CGMCC 1.1997
HldE-VP-2	GGTGGTGGTGGTGCTCGAGTTTAGCCTTTGCCGCCTTTAAT	
HldD-VP-1	AGCAAATGGGTCGCGGATCCATGATCATCGTAACTGGTGG	For the expression of $HldD_{VP}$ from *V. parahaemolyticus* CGMCC 1.1997
HldD-VP-2	GGTGGTGGTGGTGCTCGAGTTTAACTGTTTTGGATAGTTA	

Table S3 Identity comparisons of the ADP-*β*-D-*manno*-heptose synthetic enzymes from *Vibrio* strains *and E. coli.*

	$GmhA_{VC}$	$GmhB_{VC}$	$HldE_{VC}$	$HldD_{VC}$	$GmhA_{VP}$	$GmhB_{VP}$	$HldE_{VP}$	$HldD_{VP}$
$GmhA_{EC}$	79	-	-	-	78	-	-	-
$GmhB_{EC}$	-	62	-	-	-	62	-	-
$HldE_{EC}$	-	-	70	-	-	-	69	-
$HldD_{EC}$	-	-	-	74	-	-	-	87

Notes: Enzymes from *E. coli*, *V. cholerae* O1 2010EL-1786, and *V. parahaemolyticus* CGMCC 1.1997 are indicated with a subscript EC, VC, and VP, respectively.

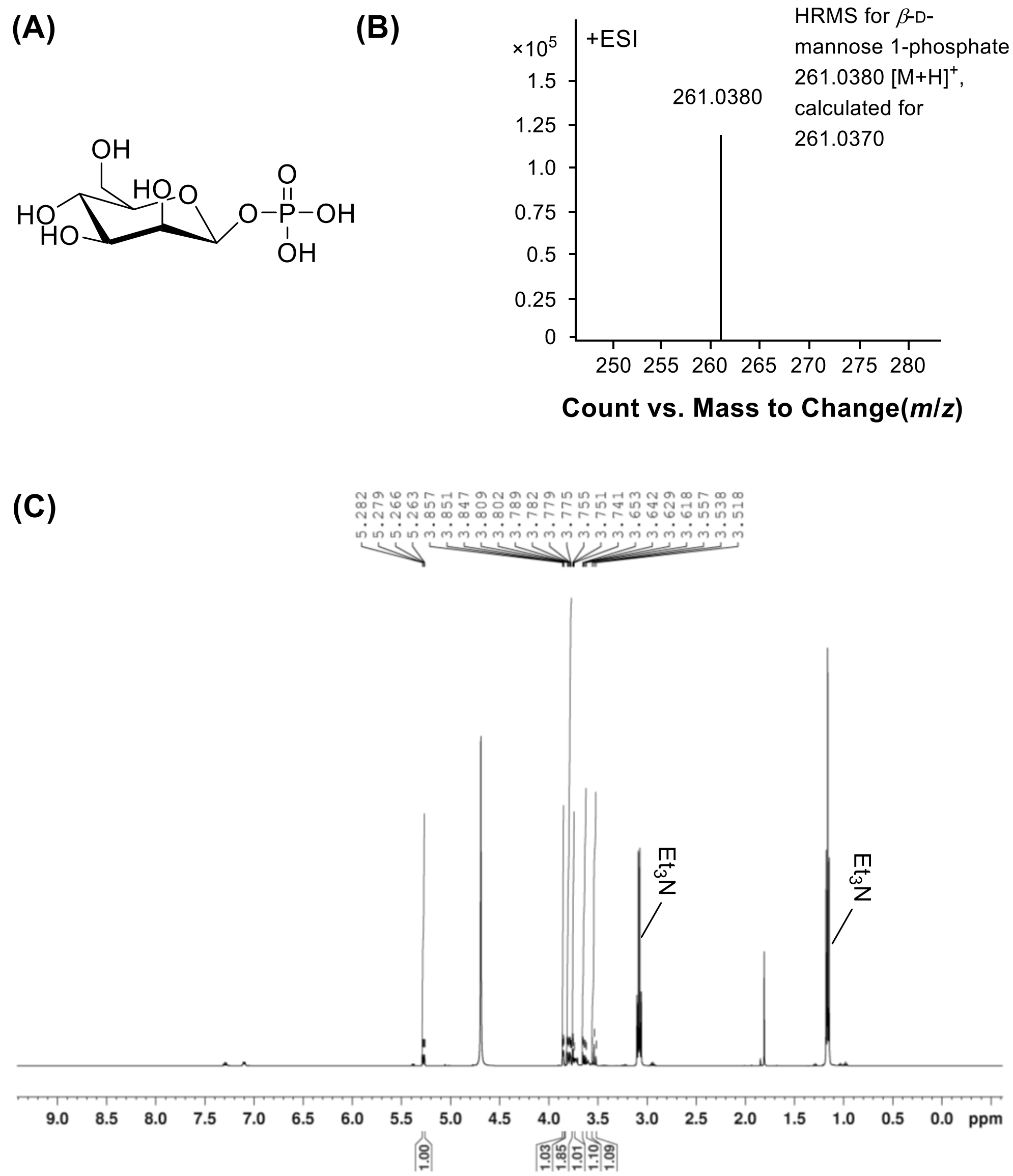

Fig. S1-1 Spectral data of *β*-D-mannose 1-phosphate. (A) Structure of *β*-D-mannose 1-phosphate. (B) HRMS spectrum of *β*-D-mannose 1-phosphate. (C) ^{1}H NMR spectrum of *β*-D-mannose 1-phosphate.

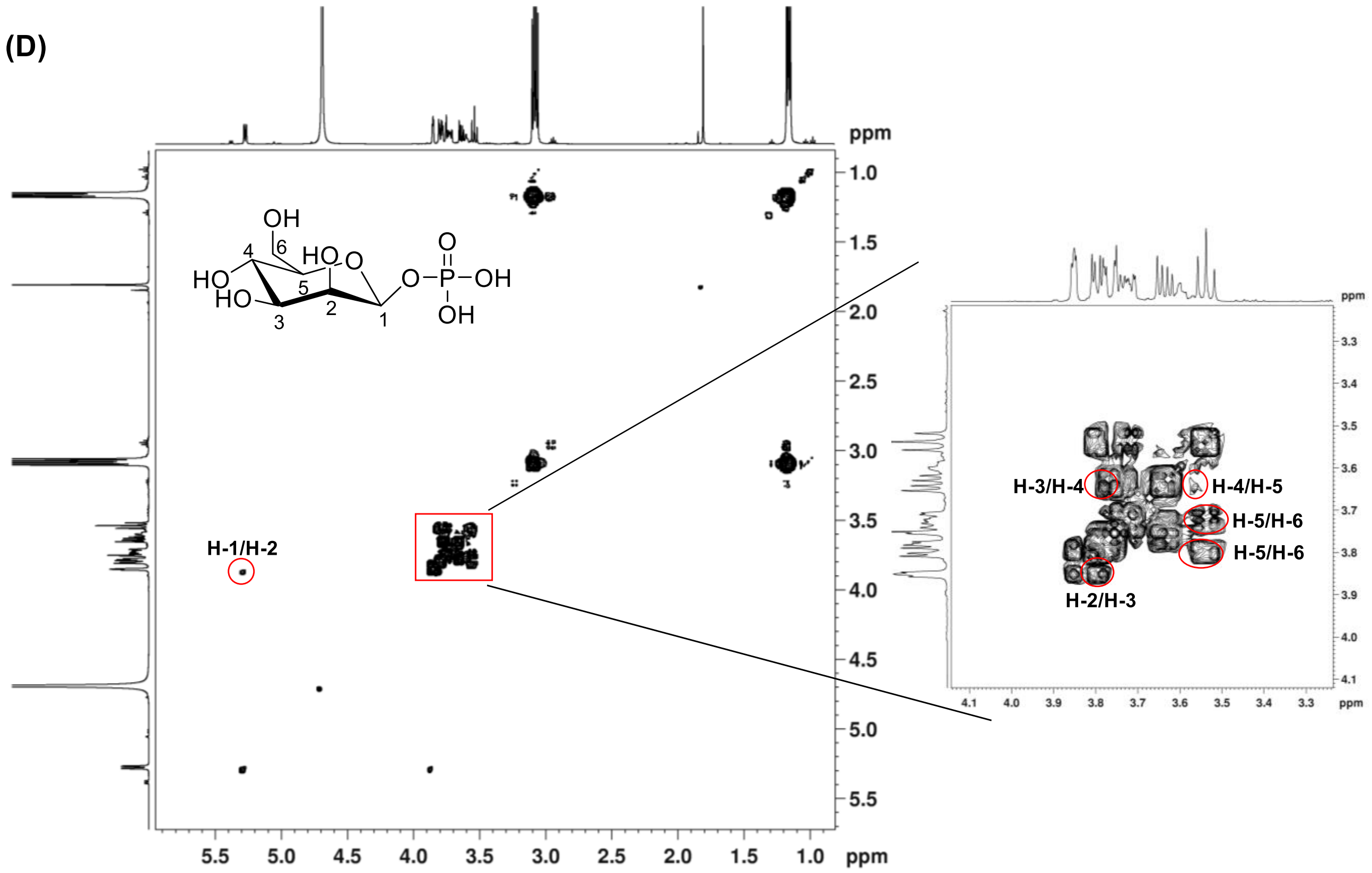

Fig. S1-2 Spectral data of *β*-D-mannose 1-phosphate. (D) ^{1}H-^{1}H COSY spectrum of *β*-D-mannose 1-phosphate. The key ^{1}H-^{1}H COSY correlations are marked on the structure of *β*-D-mannose 1-phosphate with black bold lines and at the spectrum with red circles.

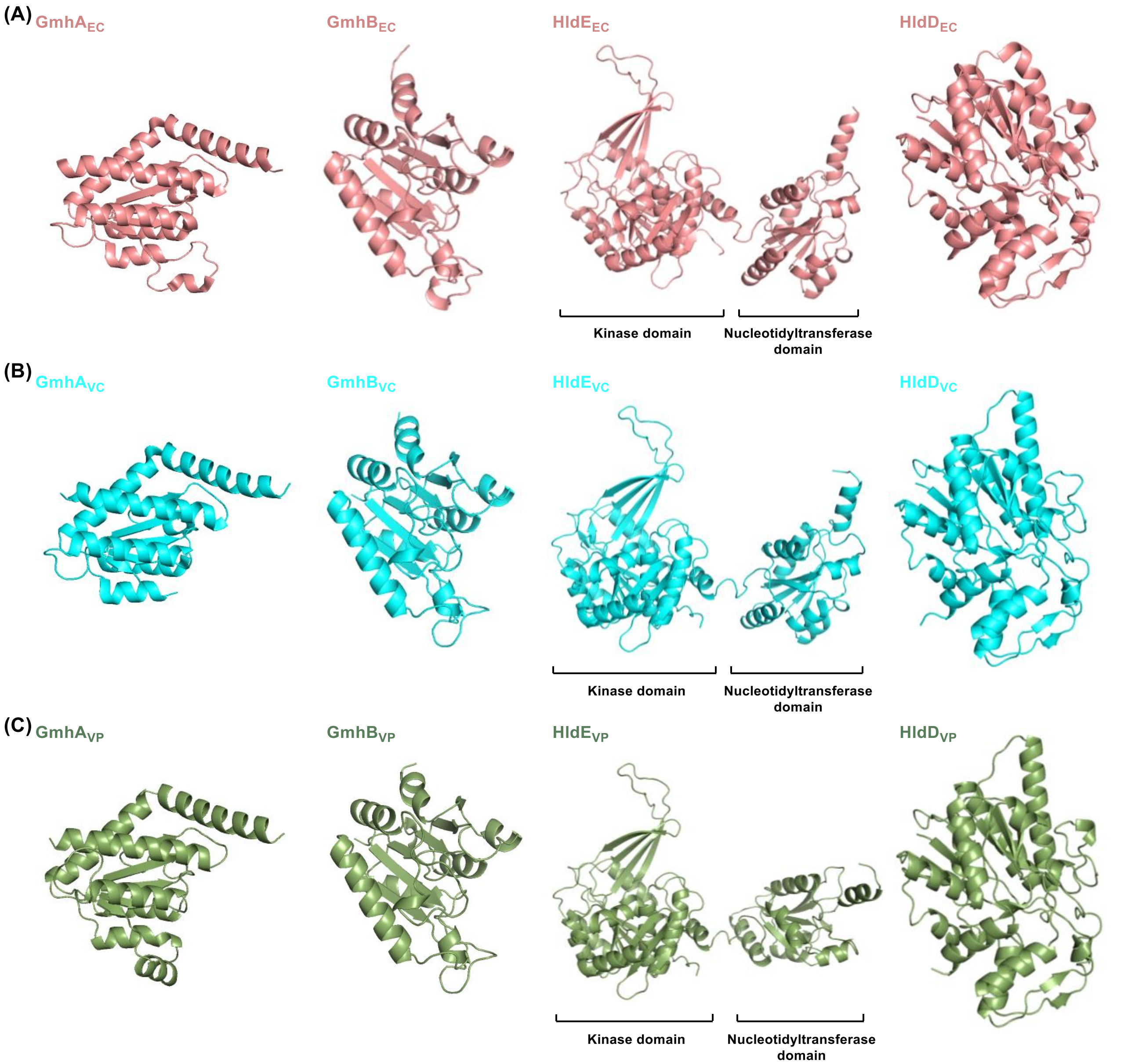

Fig. S2 The protein structures of ADP-*β*-D-*manno*-heptose biosynthetic enzymes from *E. coli* and *Vibrio* strains. (A) The crystal or predicted protein structure monomers of $GmhA_{EC}$, $GmhB_{EC}$, $HldE_{EC}$, and $HldD_{EC}$ from *E. coli*; (B) The crystal or predicted protein structure monomers of $GmhA_{VC}$, $GmhB_{VC}$, $HldE_{VC}$, and $HldD_{VC}$ from *V. cholerae* O1 2010EL-1786. (C) The predicted protein structure monomers of $GmhA_{VP}$, $GmhB_{VP}$, $HldE_{VP}$, and $HldD_{VP}$ from *V. parahaemolyticus* CGMCC 1.1997. The crystal structures $GmhA_{EC}$ (PDB code: 212W), $HldD_{EC}$ (PDB code: 1EQ2), and $GmhA_{VC}$ (PDB code: 1X94) were determined (Seetharaman et al. 2006; Taylor et al. 2008; Deacon et al. 2000) and the protein structures of the other ADP-*β*-D-*manno*-heptose biosynthetic enzymes were predicted by AlphaFold.

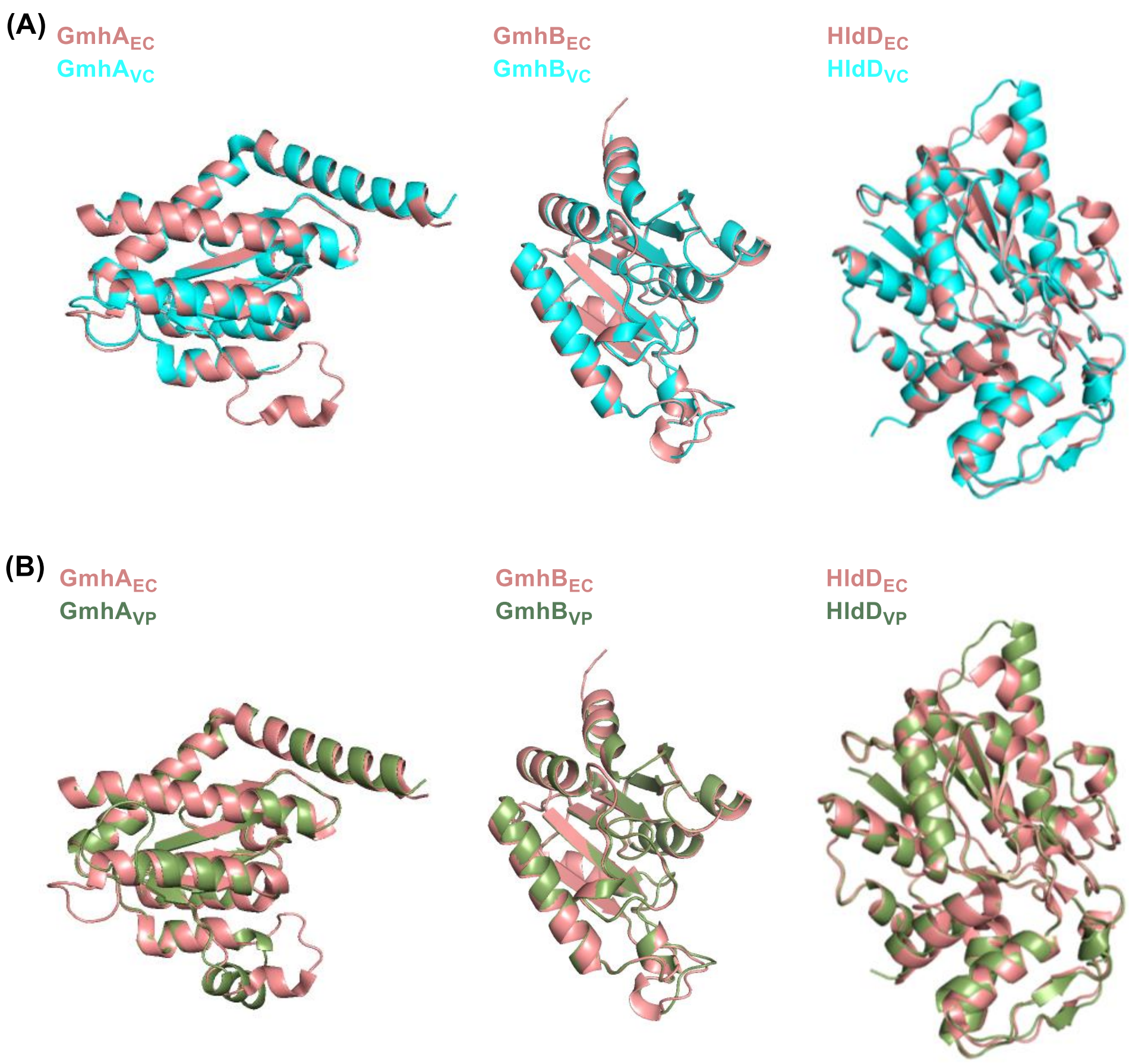

Fig. S3 Structural superimposition of the ADP-*β*-D-*manno*-heptose biosynthetic enzymes from *Vibrio* strains with *E. coli*. (A) Pairwise structural comparison of $GmhA_{VC}$ with $GmhA_{EC}$, $GmhB_{VC}$ with $GmhB_{EC}$, and $HldD_{VC}$ with $HldD_{EC}$. (B) Pairwise structural comparison of $GmhA_{VP}$ with $GmhA_{EC}$, $GmhB_{VP}$ with $GmhB_{EC}$, $HldD_{VP}$ with $HldD_{EC}$. The ADP-*β*-D-*manno*-heptose biosynthetic enzymes from *E. coli*, *V. cholerae* O1 2010EL-1786, *V. parahaemolyticus* CGMCC 1.1997 are indicated with a subscript of EC, VC, and VP, respectively.

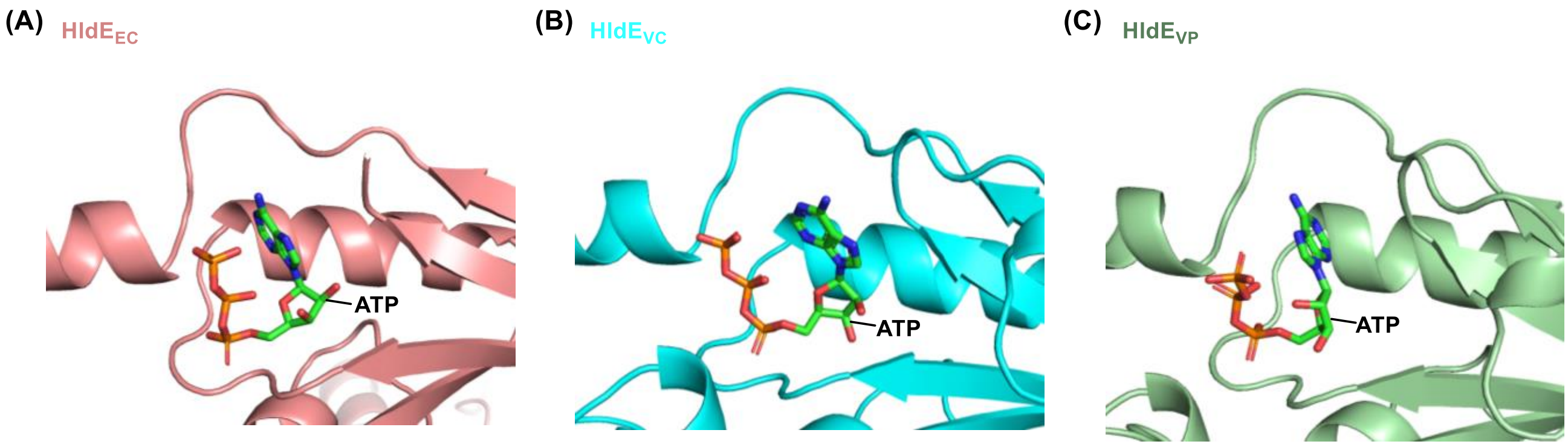

Fig. S4 The docking results of ATP in the substrate binding pockets of nucleotidyltransferase domains of HldEs. (A) The 'horseshoe' binding model of ATP in HldE$_{EC}$-ATP; (B) The 'horseshoe' binding model of ATP in HldE$_{VC}$-ATP; (C) The 'horseshoe' binding model of ATP in HldE$_{VP}$-ATP.

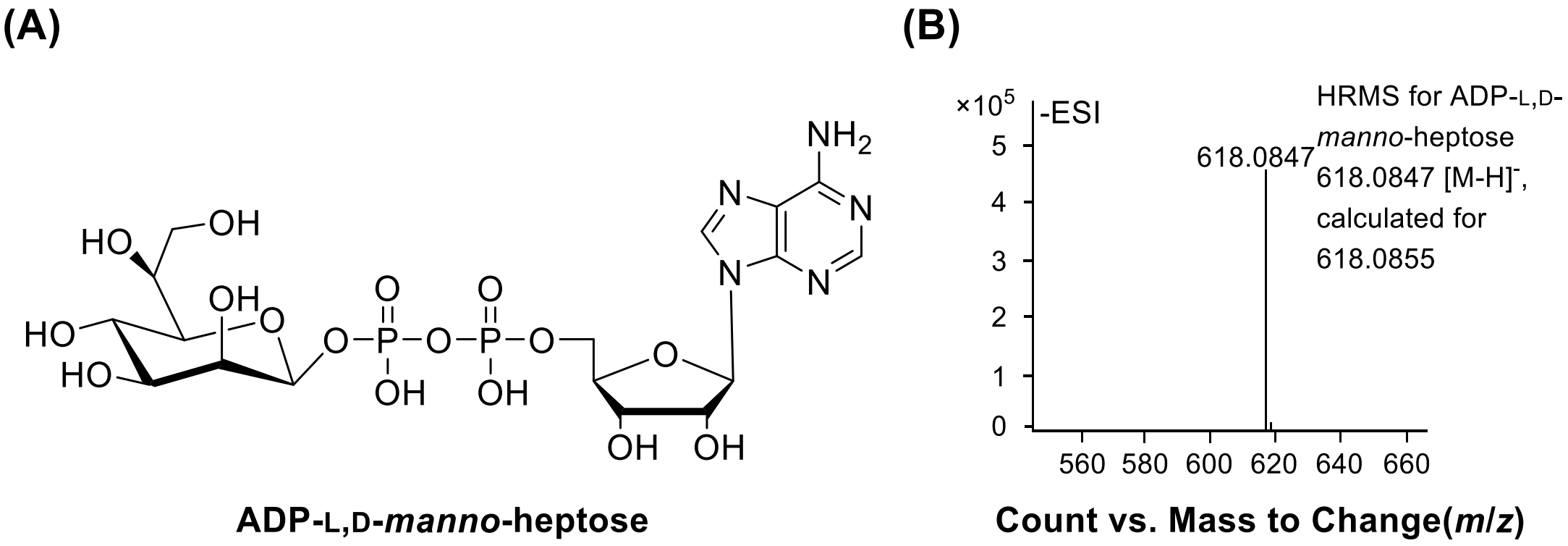

Fig. S5 HRMS data of ADP-L,D-*manno*-heptose. (A) Structure of ADP-L,D-*manno*-heptose. (B) HRMS spectrum of ADP-L,D-*manno*-heptose prepared from the $HldD_{VC}$ reaction.

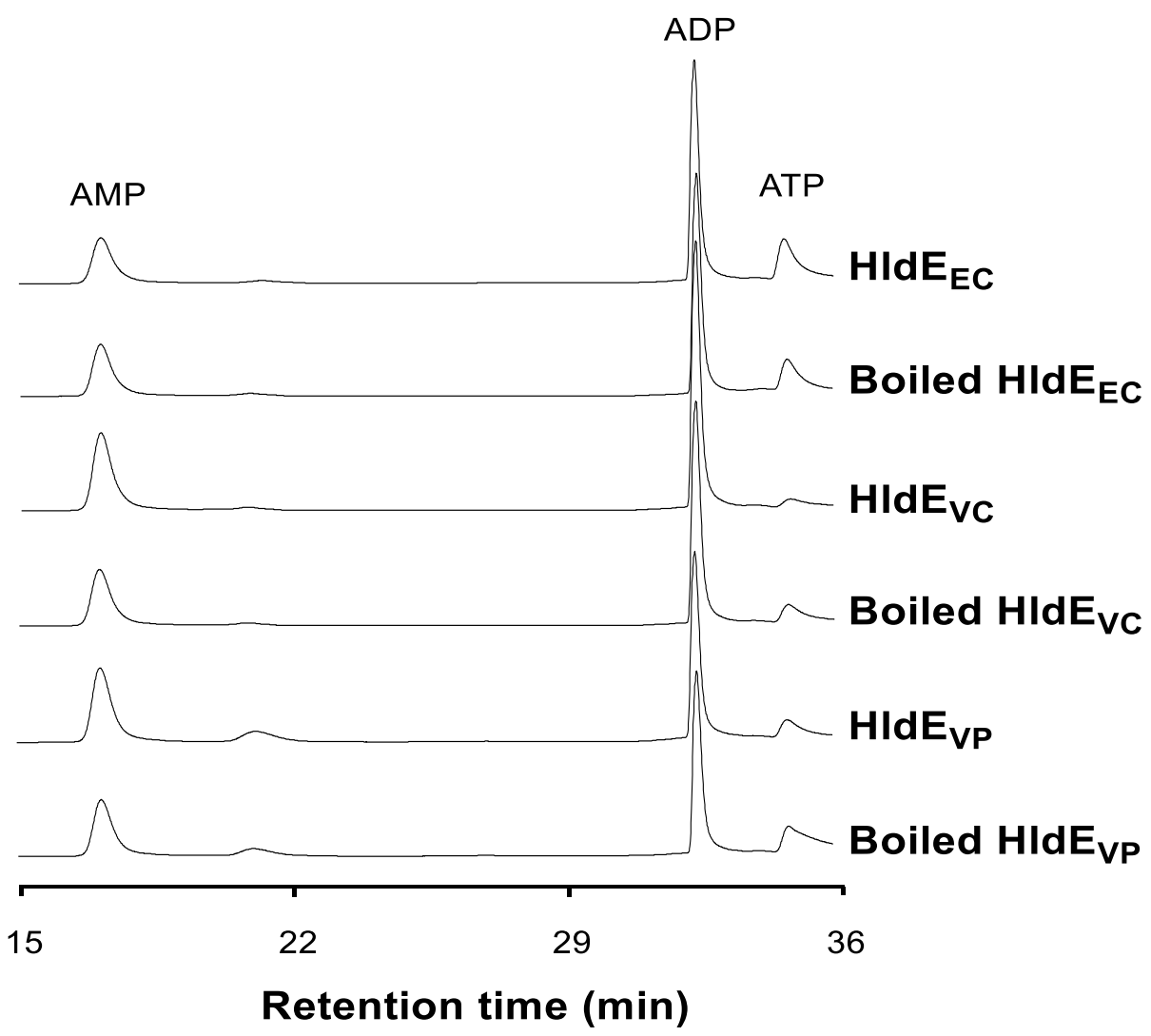

Fig. S6 HPLC profiles of the enzymatic assays catalyzed by the nucleotidyltransferase domain of HldEs using *α*-D-mannose 1-phosphate and ATP as substrates. The detection wavelength was set as 254 nm. HldE$_{EC}$, HldE$_{VC}$, and HldE$_{VP}$ are bifunctional kinase/nucleotidyltransferases from *E. coli* BL21, *V. cholerae* O1 2010EL-1786, and *V. parahaemolyticus* CGMCC 1.1997, respectively.

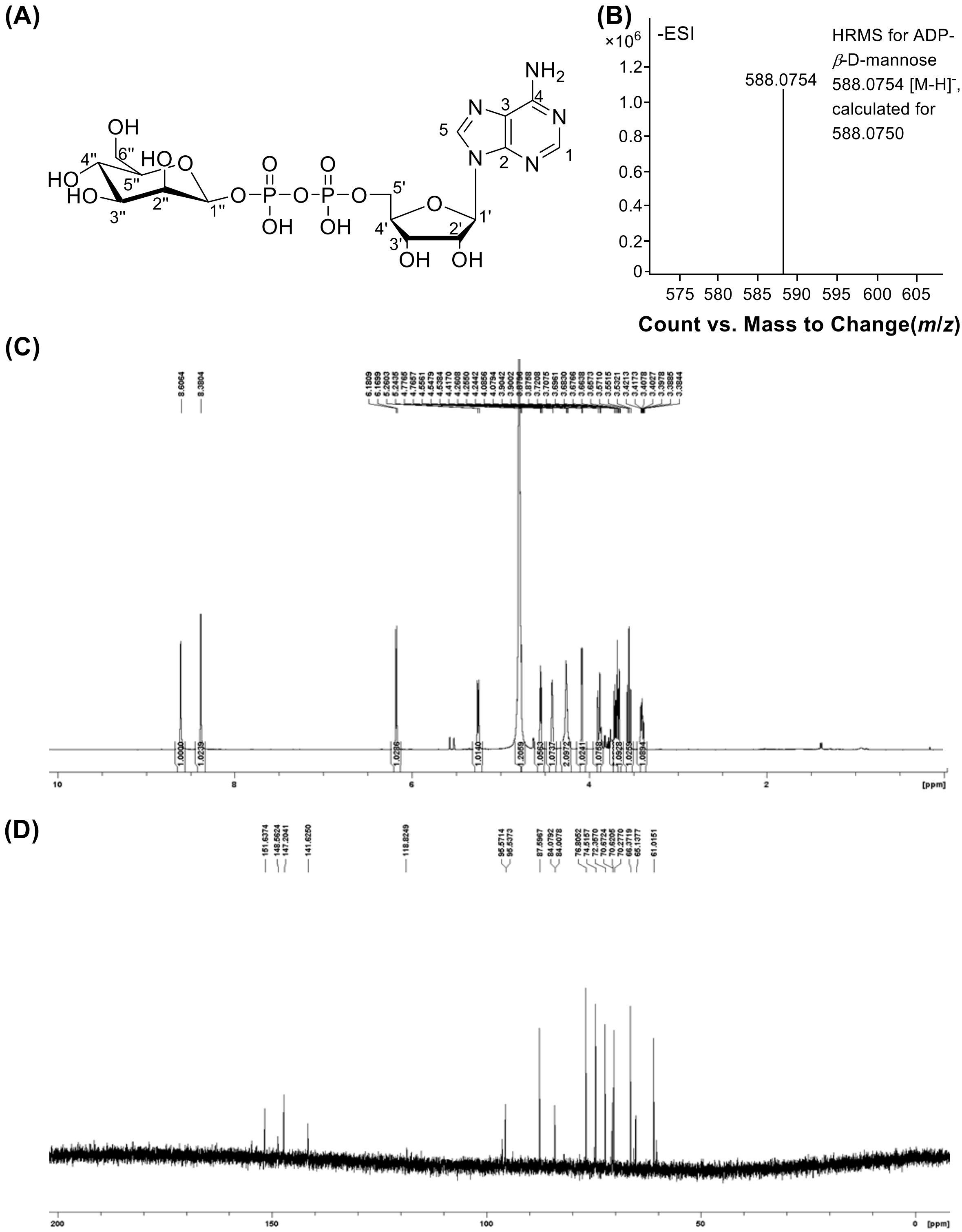

Fig. S7-1 Spectral data of ADP-*β*-D-mannose. (A) Structure of ADP-*β*-D-mannose. (B) HRMS spectrum of ADP-*β*-D-mannose. (C) ^{1}H NMR spectrum of ADP-*β*-D-mannose. (D) ^{13}C NMR spectrum of ADP-*β*-D-mannose.

(E)

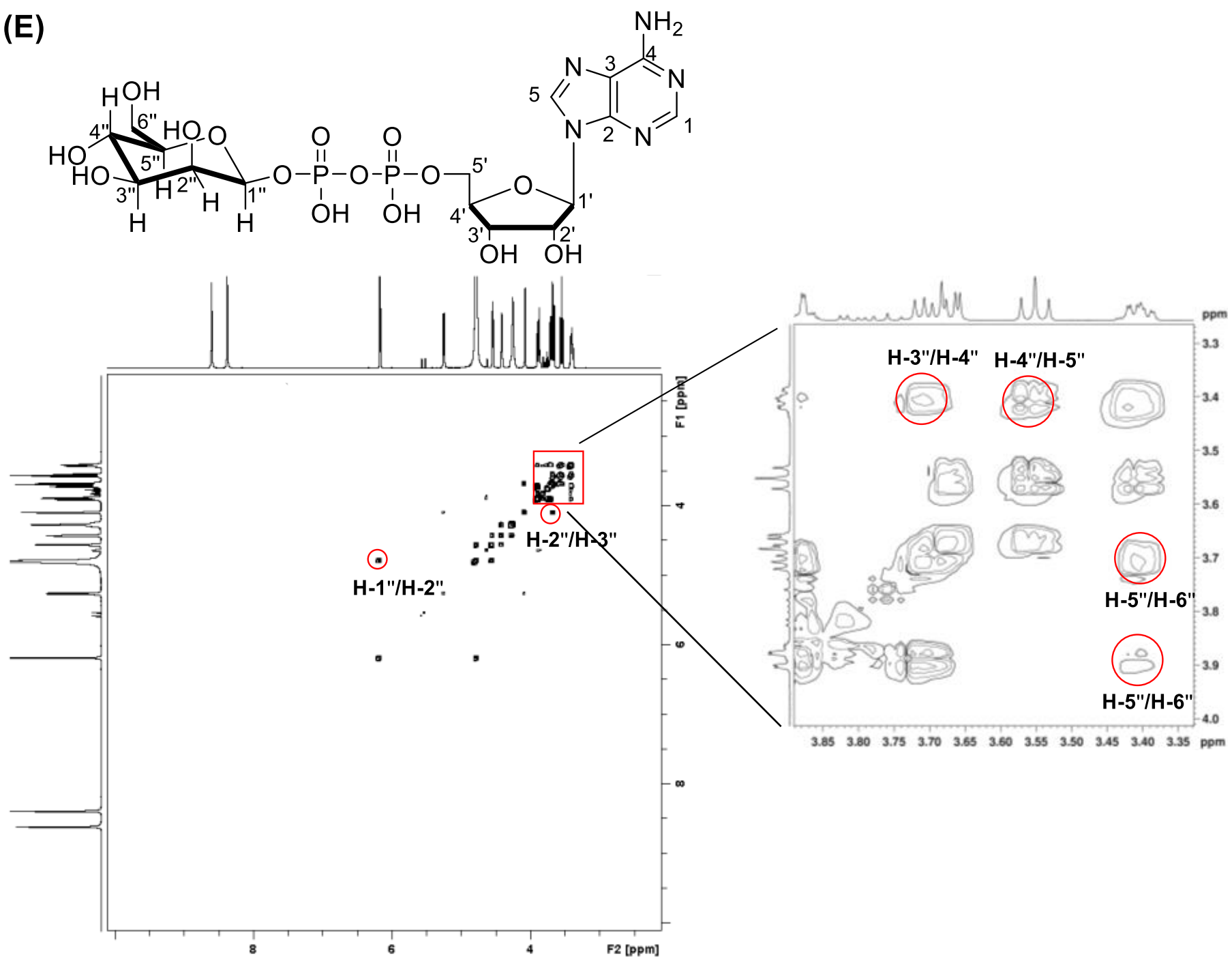

(F)

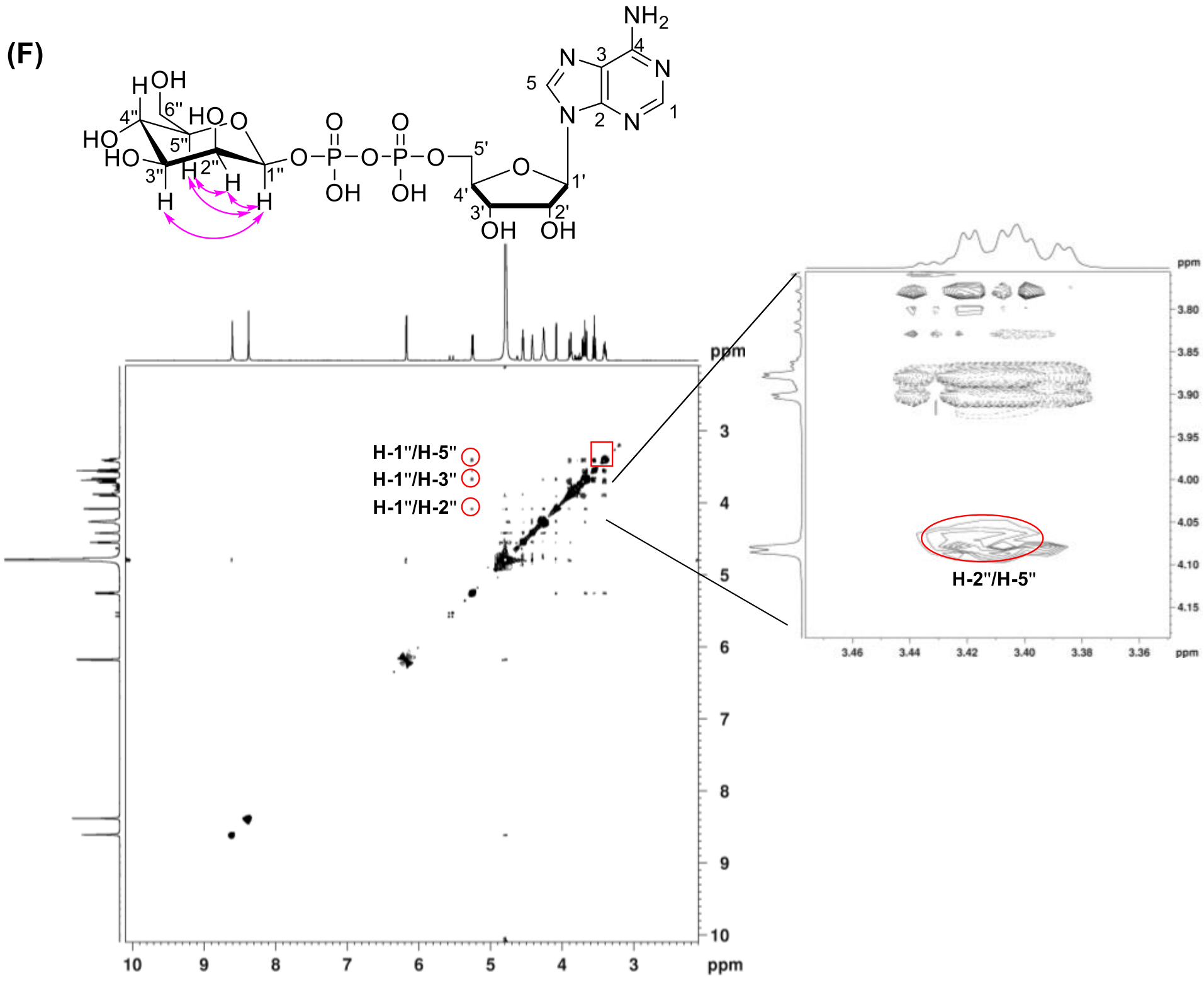

Fig. S7-2 Spectral data of ADP-*β*-D-mannose. (E) ^{1}H-^{1}H COSY spectrum of ADP-*β*-D-mannose. (F) NOE spectrum of ADP-*β*-D-mannose. The key ^{1}H-^{1}H COSY correlations are marked on the structure of ADP-*β*-D-mannose with black bold lines and at the spectrum with red circles. The key NOE correlations are marked on the structure of ADP-*β*-D-mannose with pink arrows and at the spectrum with red circles.

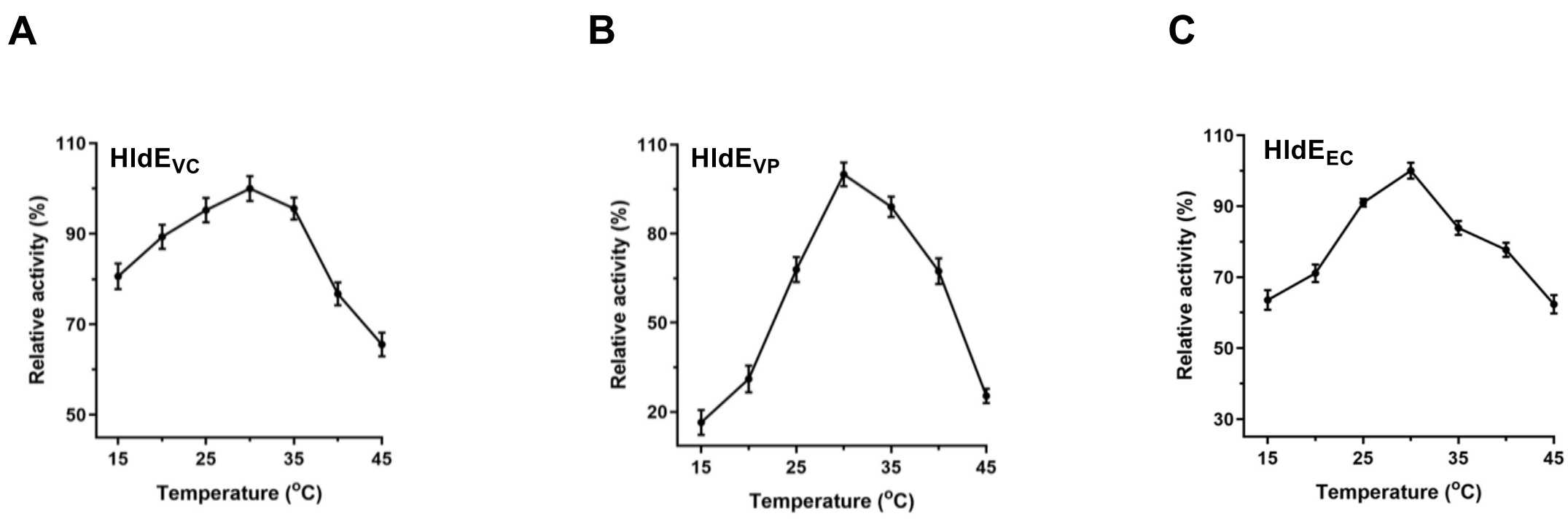

Fig. S8 Determination of the optimal temperatures of HldE$_{VC}$, HldE$_{VP}$, and HldE$_{EC}$. (A) The effect of temperature on HldE$_{VC}$ activity. (B) The effect of temperature on HldE$_{VP}$ activity. (C) The effect of temperature on HldE$_{EC}$ activity. All the reactions were performed in a 50 μL volume mixture containing 2 mM $MgCl_2$, 0.2 mM ATP, 0.2 mM *β*-D-mannose 1-phosphate, 5 μM HldE, and 0.1 U PPase at their optimal pH values (HldE$_{VC}$, pH 8.5; HldE$_{VP}$, pH 8.0; HldE$_{EC}$, pH 7.5) for 0.5 h. All the reactions were detected using the colorimetric assay. Values and error bars represented means and standard deviations of biological triplicates.

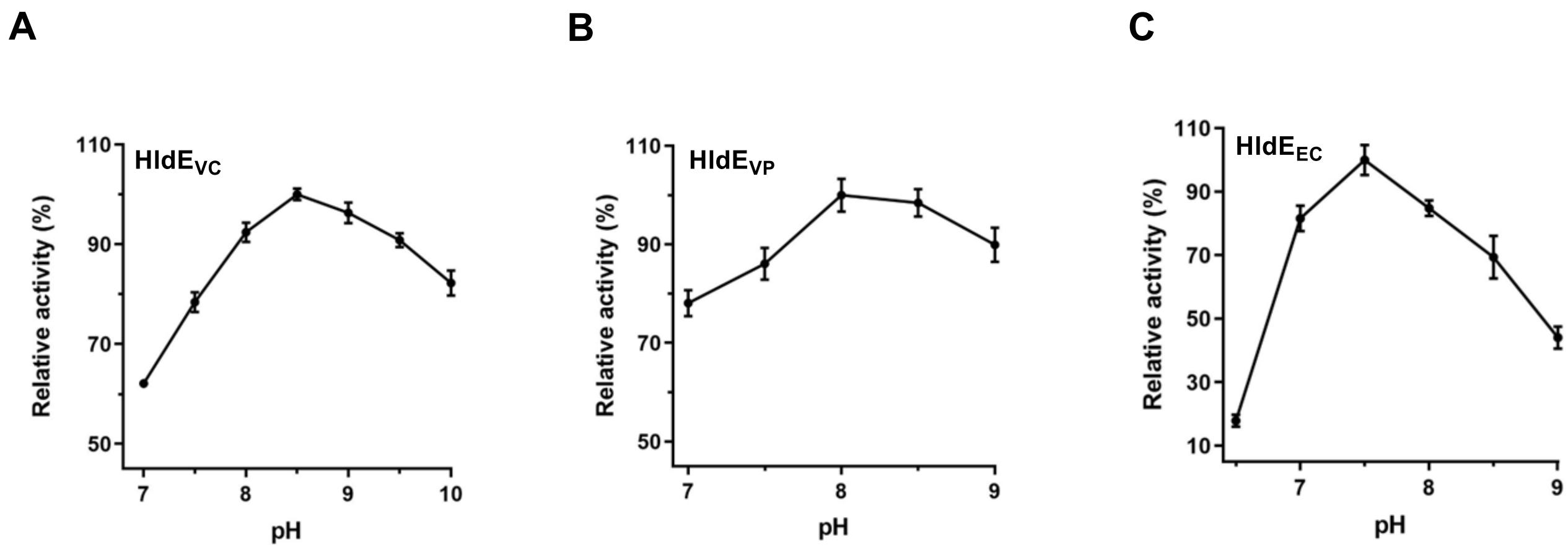

Fig. S9 Determination of the optimal pHs of HldE$_{VC}$, HldE$_{VP}$, and HldE$_{EC}$. (A) The effect of pH on HldE$_{VC}$ activity. (B) The effect of pH on HldE$_{VP}$ activity. (C) The effect pH of on HldE$_{EC}$ activity. All the reactions were performed in a 50 μL volume mixture containing 2 mM $MgCl_2$, 0.2 mM ATP, 0.2 mM *β*-D-mannose 1-phosphate, 5 μM HldE, and 0.1 U PPase at 30 °C for 0.5 h. All the reactions were monitored using the colorimetric assay. Values and error bars represented means and standard deviations of biological triplicates.

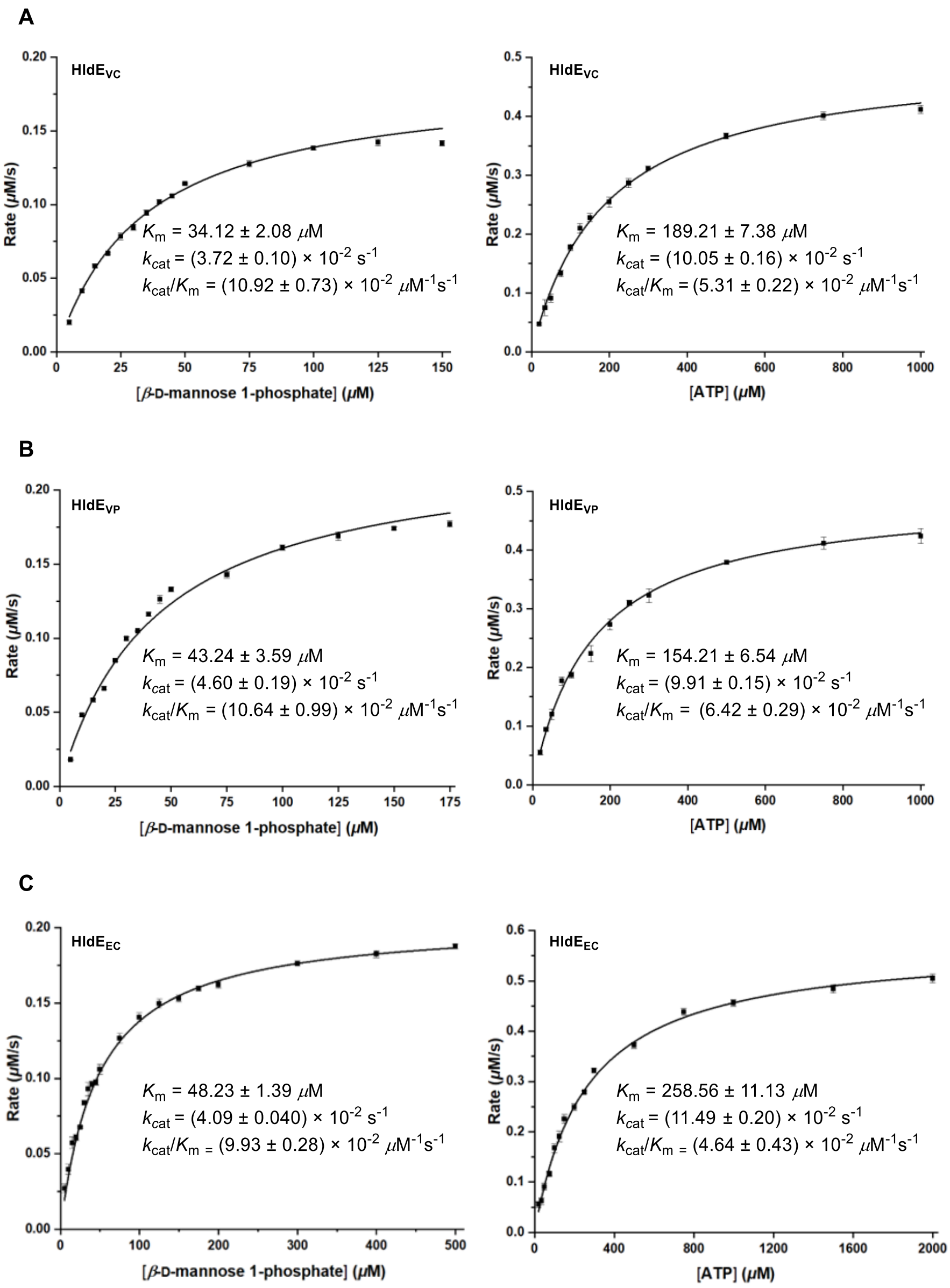

Fig. S10 The steady-state kinetic analyses of HldE$_{VC}$, HldE$_{VP}$, and HldE$_{EC}$. (A) The kinetic parameters of HldE$_{VC}$ against *β*-D-mannose 1-phosphate (left) and ATP (right). (B) The kinetic parameters of HldE$_{VP}$ against *β*-D-mannose 1-phosphate (left) and ATP (right). (C) The kinetic parameters of HldE$_{EC}$ against *β*-D-mannose 1-phosphate (left) and ATP (right). Values and error bars represented means and standard deviations of biological triplicates.

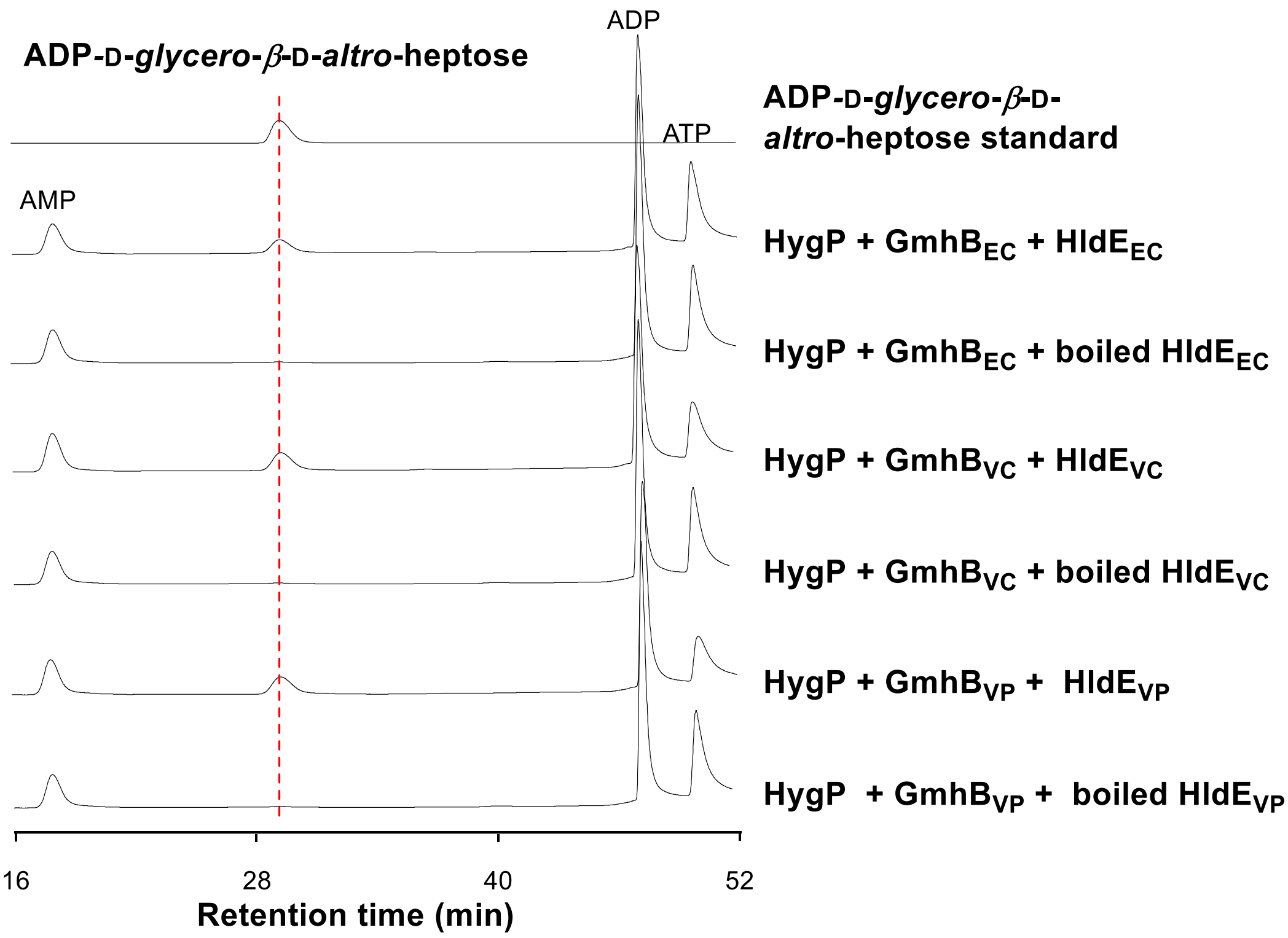

Fig. S11 HPLC profiles of the enzymatic assays of ADP-D-*glycero*-*β*-D-*altro*-heptose catalyzed by HygP and the *Vibrio* enzymes. HygP is an isomerase from *Streptomyces hygroscopicus* DSM 40578. The ADP-*β*-D-*manno*-heptose biosynthetic enzymes from *E. coli*, *V. cholerae* O1 2010EL-1786, *V. parahaemolyticus* CGMCC 1.1997 are indicated with a subscript of EC, VC, and VP, respectively. The detection wavelength was set as 254 nm.

References

Deacon, A. M., Ni, Y. S., Coleman, W. G., and Ealick, S. E. (2000). The crystal structure of ADP-L-*glycero*-D-*manno*-heptose 6-epimerase: catalysis with a twist. Structure 8(5): 453-462. doi:10.1016/s0969-2126(00)00128-3

Sabesan S, Neira S (1992) Synthesis of glycosyl phosphates and azides. Carbohydr Res 223:169-185. doi:10.1016/0008-6215(92)80015-s

Seetharaman J, Rajashankar KR, Solorzano V, Kniewel R, Lima CD, Bonanno JB, Burley SK, Swaminathan S (2006) Crystal structures of two putative phosphoheptose isomerases. Proteins Struct Funct Bioinform 63(4):1092-1096. doi: 10.1002/prot.20908

Taylor PL, Blakely KM, de Leon GP, Walker JR, McArthur F, Evdokimova E, Zhang K, Valvano MA, Wright GD, Junop MS (2008) Structure and function of sedoheptulose-7-phosphate isomerase, a critical enzyme for lipopolysaccharide biosynthesis and a target for antibiotic adjuvants. J Biol Chem 283(5):2835-2845. doi10.1074/jbc.M706163200

Tang W, Guo Z, Cao Z, Wang M, Li P, Meng X, Zhao X, Xie Z, Wang W, Zhou A, Lou C, Chen Y (2018) D-Sedoheptulose-7-phosphate is a common precursor for the heptoses of septacidin and hygromycin B. Proc Natl Acad Sci U S A 115(11):2818-2823. doi:10.1073/pnas.1711665115

Tang Y, Tang W, Wang M, Zhang Z, Chen Y (2022) A conservative distribution of tridomain NDP-heptose synthetases in actinobacteria. Sci China Life Sci 65(5):1014-1023. doi:10.1007/s11427-021-2000-2
